# Design, Synthesis, and Characterization of *N*‑Doped Carbon Dots from a Ternary System of Citric Acid, Urea, and (*E*)‑2-(2,5-Dimethoxyphenyl)methylenebutane-1,4-dioic Acid

**DOI:** 10.1021/acsomega.5c08957

**Published:** 2026-03-14

**Authors:** Vijo Poulose, Keerthivasan M. Latha, Gowtham Raj, Sabu Thomas, Józef T. Haponiuk, Reji Varghese, Thies Thiemann, Sreeraj Gopi

**Affiliations:** † Department of Chemistry, College of Science, 11239 United Arab Emirates University, Al Ain 15551, United Arab Emirates; ‡ School of Chemistry, 193159 Indian Institute of Science Education and Research (IISER) Thiruvananthapuram, Trivandrum 695551, Kerala, India; § School of Nanoscience and Nanotechnology, 29318 Mahatma Gandhi University, Kottayam 686560, Kerala, India; ∥ Department of Polymer Technology, Faculty of Chemistry Gdańsk University of Technology, Gdańsk, Pomeranian Voivodeship 80-233, Poland; ⊥ R&D Centre, Molecules Biolabs Private Limited, Koratty, Thrissur 680309, Kerala, India

## Abstract

Synthesizing carbon dots (CDs) using therapeutic compounds as precursors has emerged as a promising strategy for biomedical applications, enabling simultaneous utilization of the beneficial properties of both nanoparticles and bioactive molecules without additional modification. In this work, we report the synthesis of novel CDs (ARI-NCDs) using citric acid, urea, and (*E*)-2-(2,5-dimethoxyphenyl)­methylenebutane-1,4-dioic acid via a green, one-step hydrothermal process at 200 °C for 10 h. Control CDs prepared from citric acid and urea (U-NCDs) were synthesized identically. Comprehensive characterization by TEM, FT-IR, Raman, XRD and XPS confirmed nitrogen-doped, oxygen-rich surfaces and successful aryl functionalization. ARI-NCDs exhibited bright, nearly excitation-independent blue emission and a higher quantum yield than U-NCDs. TGA revealed the enhanced thermal stability of ARI-NCDs compared with U-NCDs. Zeta potential measurements across pH 4–10 indicated a single well-defined negative surface charge for ARI-NCDs at neutral pH, consistent with superior colloidal stability and dispersion homogeneity, while U-NCDs displayed charge heterogeneity. In vitro assays using HeLa cells demonstrated efficient cellular internalization of ARI-NCDs and low cytotoxicity, highlighting their suitability for live cell imaging and other biomedical applications. Additionally, ARI-NCDs displayed pronounced antibacterial activity against both Gram-positive and Gram-negative bacteria with larger inhibition zones and lower minimum inhibitory concentrations relative to U-NCDs, attributable to the functional groups and structural attributes imparted by the aryl itaconic acid precursor. These findings underscore the versatility of ARI-NCDs for multifunctional biomedical applications, combining imaging capability with antimicrobial efficacy.

## Graphical abstract

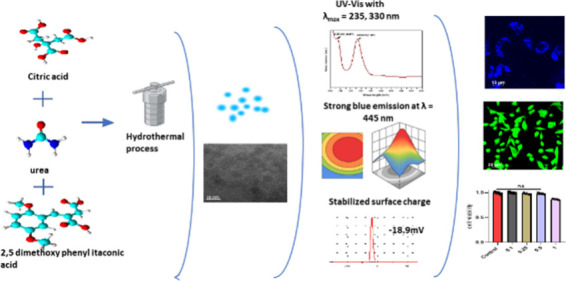

## 1 Introduction

Nanomaterials with multimodal properties have garnered significant attention in recent years, particularly for their potential in biomedical applications such as imaging and biosensing probes.^1^ The unique physicochemical characteristics of nanoparticles (NPs)—including their small size, shape, surface chemistry, and surface charge—play a crucial role in their cellular uptake and overall functionality.^2^ Their properties make them highly suitable for targeted drug delivery, as well as diagnostic and therapeutic applications.^3^ Continuous development of such nanomaterials is essential for advancing precision medicine and enhancing treatment outcomes.

Among the different types of NPs, carbon dots (CDs) have gained increasing interest due to their unique and advantageous properties, such as high photostability^4^ and quasi-spherical shape^5^ with a size of typically less than 10 nm.^6^ The presence of polar functional groups on their surface endows the CDs with a high degree of hydrophilicity,^7^ facilitating their ability to traverse cellular membranes, advantageous for their use in drug delivery, tissue engineering, bioimaging, and other medical applications.^8^ In addition, many CDs possess a large surface area, low cytotoxicity, and excellent biocompatibility, all traits suitable for their medicinal use.^9^ Also, CDs exhibit remarkable optical properties, including a multicolor emission profile.^10^ The fluorescent properties of CDs are especially advantageous for real-time drug-delivery tracking,^11^ enabling precise monitoring and improving the effectiveness of therapeutic interventions.^12^

Among the various synthesis methodologies of CDs, the hydrothermal approach has emerged as a widely adopted technique due to its simplicity, environmental friendliness, and ability to produce high-quality carbon-based nanomaterials.^13^ A commonly adopted precursor combination for CD synthesis consists of citric acid (CA) and urea in a 1:2 molar ratio, where CA serves as the carbon source, providing the carbon framework and multiple functional groups for carbonization, while urea acts as a nitrogen donor, facilitating nitrogen doping.^14^ Both CA and urea can provide surface functional groups. The carbonization process concomitant with the incorporation of nitrogen leads to high photoluminescence quantum yields, critical for biological applications.^15^ The resulting CDs typically exhibit bright blue fluorescence under UV light and are rich in amino, hydroxyl, and carboxyl groups, which contribute to their excellent solubility and cellular uptake, making the CDs promising candidates for biological applications.^16^ To further improve the fluorescence intensity and targetability, ternary precursor systems have been explored.^17,18^ For example, incorporating thiourea alongside CA and urea allows for dual N/S doping, enhancing emission properties^19^ and introducing surface defect states beneficial for fluorescence and deep-tissue imaging. In other examples, ternary precursor systems are used to tune optical properties as well as introduce a selective interaction of the CD with the target molecules to be imaged.^20^ Ternary or more complex precursor systems are also employed to synthesize CDs with biological properties—such as with antimicrobial, anticancer, antioxidant, and anti-inflammatory activities—where physiologically active moieties are integrated into the CDs themselves, providing therapeutic effects without the need for additional drug loading.^21,22^ Alternatively, these effects can be further enhanced through additional drug loading, where in the CDs function as an efficient drug-delivery platform.

A significant advancement in CD design is the development of materials that exhibit an excitation-independent photoluminescence (PL), a feature highly desirable for precision optical applications. A typical example was reported by Wen and Yin^23^ with the successful synthesis of blue and green emitting CDs, which exhibited fixed emission peaks, irrespective of the excitation wavelength. This behavior was attributed to the homogeneous π-conjugated carbon core structures, which govern the optical properties, and to the presence of surface functional groups, such as amide and imine functions that enable distinct vibrational relaxation pathways. Notably, these CDs exhibited large Stokes shifts (up to 230 nm), high quantum yields (up to 69.3%), and narrow full-width at half-maximum values. Such characteristics are particularly beneficial in fluorescence-based bioimaging, where stable single-color emission reduces spectral overlap and improves signal-to-noise ratios. Moreover, the substantial Stokes shifts minimize reabsorption and excitation interference, thereby improving detection sensitivity in complex biological environments.^24^ Therefore, the preparation of CDs with such properties remains a highly interesting target.

Itaconic acid, a byproduct^25^ of the tricarboxylic acid cycle, the second stage of cellular respiration, exists as its dianion, known as itaconate, at physiological pH above pH 7. It is nontoxic to humans and demonstrates anti-inflammatory properties in the body.^26^ Itaconate influences cells by modulating various response-regulating pathways. Specifically, it activates the OXGR1 receptor (GPR99),^27^ which acts as a receptor to α-ketoglutarate, an intermediate in the tricarboxylic acid cycle, and leukotriene E4, a cysteinyl leukotriene involved in inflammation.^28^

In addition to its anti-inflammatory effects, itaconate, along with its mono- and diesters, exhibits antimicrobial and antiviral properties.^29,30^ Itaconic acid and its derivatives have also been shown to have anticancer actions.^31^ Previous studies demonstrated that arylated itaconic acid derivatives, including­(*E*)-2-(2,5-dimethoxyphenyl)­methylenebutane-1,4-dioic acid, exhibit notable antimicrobial activities.^32^

Itaconic acid and its derivatives serve as versatile platforms for diverse chemical transformations.^33,34^ Itaconic acid is a good Michael acceptor, while the arylated derivatives are expected to undergo a mixture of 1,2- and 1,4-addition reactions. The olefinic group is also prone to pericyclic reactions. For 2,5-dimethoxyphenyl methylenebutane-1,4-dioic, the methoxy groups act as electron donors, enriching the aromatic ring enough to enable Friedel–Crafts-type alkylation and acylation reactions. Under the conditions of the hydrothermal reaction to CDs, (*E*)-2-(2,5-dimethoxyphenyl)­methylenebutane-1,4-dioic acid would be expected both to build into the CD structure and to attach itself, including covalently, to the CD surface. It was anticipated, in this manner, that (*E*)-2-(2,5-dimethoxyphenyl)­methylenebutane-1,4-doic acid would impart some of its biological properties to the resulting CDs while also enhancing CD-cell interactions.

Based on these considerations, this study aims to develop new biocompatible and structurally distinct CDs through a green, one-step hydrothermal synthesis using CA, urea, and the aromatic itaconic acid derivative (*E*)-2-(2,5-dimethoxyphenyl)­methylenebutane-1,4-dioic acid as precursors. Synthesized via a one-pot ring-opening–Wittig olefination–hydrolysis sequence from 2,5-dimethoxybenzaldehyde and (triphenylphosphoranylidene)­succinic anhydride, the unique precursor (*E*)-2-(2,5-dimethoxyphenyl)­methylenebutane-1,4-dioic acid is expected to introduce functionally rich aromatic groups onto the CD surface, yielding ARI-NCDs with enhanced optical emission and distinct surface functionalities—such as carboxyl and methoxy groups—that are known to improve biocompatibility and facilitate cellular uptake. A comparative preparation of control CDs from the binary (urea–CA) system is to enable clear delineation of the role of the aryl itaconic acid in tailoring the surface chemistry.

The novelty of this work stems from the use of a new ternary system incorporating a green multifunctional precursor for the sustainable synthesis of CDs with tunable surface properties. This approach is to offer a simple, scalable route to fluorescent and biocompatible CDs, well-suited for bioimaging and therapeutic applications, representing an alternative to the more conventional binary CD systems in both design and biomedical potential.

## 2 Experimental Section

### 2.1 Materials

Citric acid monohydrate and urea were sourced from Sigma-Aldrich. Dialysis tubing with a molecular weight cutoff (MWCO) of 1000 Da was purchased from Spectrum Laboratories, Inc. Milli-Q water (resistivity: 18.2 MΩ·cm; pH: 6.5 ± 0.3 at 20.0 ± 0.5 °C) was obtained from a Milli-Q water purification system (Millipore Sigma) and used as the solvent for the preparation, purification, and analysis of CDs. All chemicals were used as received without further treatment. The HeLa cells were obtained from NCCS Pune, cultured in Dulbecco’s modified eagle medium (DMEM) media supplemented with 10% fetal bovine serum (FBS) and 1% penicillin-streptomycin and cultured at 37 °C in a 5% CO_2_ incubator. The media, FBS, and antibody were purchased from Gibco-Thermo Fisher Scientific. Calcein-AM viability dye, lysotracker, propidium iodide (PI), and methyl thiazolyl tetrazolium (MTT) reagent were purchased from Thermo Fisher Scientific.

### 2.2 Synthesis of ARI-NCDs Using (*E*)-2-(2,5-Dimethoxyphenyl)­methylenebutane-1,4-dioic Acid

The ARI-NCDs were synthesized via a one-step hydrothermal reaction, as illustrated in Scheme 1. Briefly, CA (0.2 g, 1.0 mmol) and urea (0.4 g, 6.7 mmol) were dissolved in water (Milli-Q, 5 mL). To this solution was added (*E*)-2-(2,5-dimethoxyphenyl)­methylenebutane-1,4-dioic acid (15 mg, 0.06 mmol). The mixture was stirred at RT for 25 min. Then, the resulting solution was transferred to a Paar hydrothermal autoclave (50 mL) and heated at 200 °C for 10 h in a carbonite muffle furnace. The resulting solution was centrifuged at 10,000 rpm for 10 min, followed by filtration through a filter (0.22 μm pore size), and dialyzed (MWCO:1000 Da) against Milli-Q water for 48 h, where the water was replaced every 6 h. The resulting solution was subjected to controlled evaporation *in vacuo* at 50 °C using a rotavapor. The residue was vacuum-dried at 50 °C for 12 h to provide the product in a powder form.

**Scheme 1:**
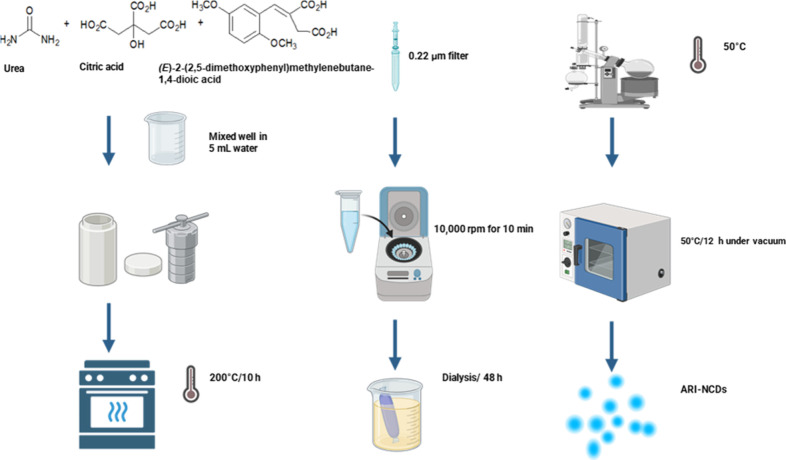
Procedure for the Hydrothermal Synthesis of ARI-NCDs (Created with BioRender.com)

### 2.3 Control CDs

For comparative purposes, control CDs (U-NCDs) were synthesized identically but using only CA (0.2 g, 1.0 mmol) and urea (0.4 g, 6.7 mmol) in Milli-Q water. Workup and purification followed the protocol described in Section 2.2.

### 2.4 Characterization Techniques

The size and morphology of the CDs were characterized by **transmission electron microscopy (TEM)** (FEI Tecnai-TF30-TEM) operated at 300 kV, for which the samples were drop-casted on a 200-mesh carbon-coated copper grid (Ted Pella, Inc.) and dried overnight at room temperature in a desiccator. **Fourier transform infrared (FT-IR) analysis** of all starting materials and CD samples were carried out using a Thermo Nicolet Nexus 670 infrared spectrometer. The FT-IR spectra were recorded in the range of 4000–500 cm^–1^ with 64 scans. The spectra were acquired using KBr pellets (1:300 weight ratio). **X-ray diffraction (XRD)** patterns of the CD samples were recorded using a Rigaku Miniflex diffractometer (Japan) with Cu Kα radiation (λ = 1.54 Å) operated at voltage 45 kV and 40 mA. The diffraction data were collected over a 2θ range of 10–70°.

**X-ray photoelectron spectroscopy (XPS)** measurements were conducted with a Thermo Scientific Nexsa G2 at room temperature in an ultrahigh vacuum system equipped with an electron analyzer detector operating at a base pressure of 10^–10^ mbar. Core level photoemission spectra were acquired in the emission mode using an Al Kα X-ray source assisted by a low-energy electron flood gun. The survey spectrum was obtained with a standard two-scan procedure, while high-resolution C 1s, N 1s, and O 1s spectra were acquired with more than five scans. The spectral regions were then analyzed and compared based on their binding energy values.

**Raman spectra** were collected on a Renishaw inVia Qontor Raman microscope by irradiating the samples sprinkled on a stainless-steel slide at the excitation wavelengths λ = 402 and 532 nm. **Thermogravimetric analysis (TGA)** was performed using a Mettler TGA 2 instrument. TGA was performed under a N_2_ atmosphere (flow rate 20 mL min^–1^) with a ramp of 5 °C/min up to 750 °C. **Field emission scanning electron microscopy** equipped with **energy-dispersive spectroscopy (EDX)** was used to analyze the carbonaceous residue obtained at 750 °C after TGA analysis. The measurements were conducted using a ThermoFisher Quattro S microscope in a high-vacuum mode with an Everhart–Thornley detector for imaging to enhance conductivity and imaging resolution. The sample was coated with a gold (Au) target prior to analysis.

**UV–vis spectra** were measured in the absorbance mode from λ = 200–600 nm with an Agilent Cary-60 UV spectrophotometer. All measurements were taken on samples diluted with Milli-Q water, where the same quartz cuvette was used for all measurements. **Fluorescence spectroscopy** measurements were performed by using a Horiba Duetta fluorescence and absorbance spectrometer. The data were collected in an excitation/emission range of λ = 300–750 nm. All sample measurements were carried out with Milli-Q water as the diluent.

The **PL quantum yield (QY)** was measured by using a Horiba Duetta spectrometer. A freshly prepared quinine sulfate solution in sulfuric acid (0.1 M, refractive index *n* = 1.33) was used as the reference standard. Quinine sulfate has a QY of 54%. Concentrations of both the standard and CDs were adjusted to ensure that at λ = 350 nm, their absorbance values in the UV spectrum were controlled between 0.01 and 0.1. The PL emission spectra of the standard and CDs were recorded upon excitation of the materials at λ = 350 nm. The averages of absorbance and PL spectral integrated areas were substituted into eq 1:

$$\Phi =\Phi_{R}\times (I/I_{R})\times (A_{R}/A)\times ({\overset{´}{\eta }}^{2}/{{\overset{´}{\eta }}^{2}}_{R})$$ 1

In this equation, Φ represents the QY, *I* indicates the integrated area under the PL curve, *A* denotes the absorbance at λ = 350 nm, and ή represents the refractive index. Subscript _R_ stands for the reference standard.

The **zeta potential** (mV) was measured on a Malvern Zetasizer Nano ZS instrument. The CD samples were dispersed in Milli-Q water, and a Zetasizer cell was used at 25 °C after a 120 s temperature equilibration time.

### 2.5 Cellular Uptake and Subcellular Localization Studies

HeLa cells were seeded in μ-Slide 8-well slides at a seeding density of 1000 cells per well and cultured with DMEM containing 10% FBS at 37 °C in 5% CO_2_ for 24h. After confluency reached up to 70%, the cell culture medium was replaced with the medium containing CDs, and the cells were further incubated for 12 h. After 12 h of incubation, the medium was replaced with fresh DMEM containing 10 μL of Lysotracker deep red (0.35 mg/mL). After 15 min of incubation, the medium was removed, and the cells were washed three times with PBS and imaged using a Nikon Eclipse Ti confocal laser scanning microscope.

### 2.6 Cytotoxicity Assessments

**Cell viability** was studied by an MTT assay. For this, HeLa cells were seeded in 96-well plates at 100 cells per well and cultured for 24 h until reaching confluency. Then, the cells were treated with CDs (0.1–1 mg/mL). After incubation for 24 h, the medium was replaced with fresh culture medium, and 20 μL of 5 mg/mL MTT solution was added. The cells were incubated for another 4 h, and 100 μL of DMSO was added to solubilize the formazan crystals. The absorbance at λ = 565 nm was measured using a microplate reader to evaluate the cytotoxicity.

The **cytotoxicity** of CDs was analyzed using a live/dead cell assay. For this, HeLa cells were obtained from NCCS Pune, cultured in DMEM media, seeded in μ-Slide 8-well slides at a seeding density of 1000 cells per well, and cultured with DMEM containing 10% FBS at 37 °C in 5% CO_2_ for 24 h. After confluency had reached up to 70%, the cell culture medium was replaced with the medium containing CDs, and the cells were further incubated for 24 h. After the incubation period was over, the cells were washed with PBS, and calcein AM (1 μM) and PI (10 μL from 10 μg/mL) in fresh medium were added and incubated for 30 min. The plates were further washed with 1 X PBS and then imaged using confocal laser scanning microscopy (CLSM).

### 2.7 Antimicrobial Activity and MIC Assay

All bacterial strains were procured from the microbial collection center (IMTECH, Chandigarh, India) including Gram-positive species—*Staphylococcus aureus* MTCC 3160, *Staphylococcus lentus* MTCC 2292, *Bacillus cereus* MTCC 6629, and *Bacillus subtilis* MTCC 1305—and Gram-negative species— *Pseudomonas aeruginosa* MTCC 1748, *Pseudomonas putida* MTCC 2492, *Escherichia coli* MTCC 1554, and *Klebsiella pneumoniae* MTCC 3040. Single bacterial colonies were inoculated into nutrient broth (50 mL in a 150 mL flask) and incubated at 37 °C for 8–12 h to prepare active cultures. Streptomycin (10 μg mL^–1^) was used as a positive control. Test samples were used directly or diluted with sterile water as required (refer to Table S1 for working concentrations).

For the Agar well diffusion assay, sterilized nutrient agar (121 °C, 15 lbs, 15 min) was dispensed into Petri plates and surface -inoculated with bacterial cultures using sterile swabs. Wells were bored into the solidified agar and charged with 100 μL of either the test sample or control, followed by incubation at 37 °C for 18–24 h. Antibacterial activity was evaluated by measuring the diameter of inhibition zones. Minimum inhibitory concentration (MIC) was determined using broth microdilution with 2-fold serial dilutions of the test samples in the nutrient broth. Standardized bacterial inoculum was added, and the plates were incubated at 37 °C for 18–24 h.The MIC for each strain was defined as the lowest sample concentration resulting in no visible bacterial growth (see Table S2 for MICs).

## 3 Results and Discussion

### 3.1 Design of ARI-NCDs and U-NCDs

We successfully synthesized (*E*)-2-(2,5-dimethoxyphenyl)­methylenebutane-1,4-dioic acid in our previous study from 2,5-dimethoxybenzaldehyde and (triphenylphosphoranylidene)­succinic anhydride via a one-pot ring-opening Wittig olefination and hydrolysis reaction (Scheme S1). The product was characterized by FT-IR, ^1^H and ^13^C NMR, (Figure S1a,b), and mass spectrometry. It was found to exhibit biological activity against two Gram-positive (*Staphylococcus aureus* and *Staphylococcus lentus*) and two Gram-negative bacterial strains ( *Pseudomonas aeruginosa* and *Pseudomonas putida* ) (Figure S2a–d).

Both ARI-NCDs and U-NCDs were synthesized via a hydrothermal method under the same reaction conditions. For comparative analysis, U-NCDs were synthesized from CA (1.0 mmol) and urea (6.7 mmol) without the addition of an aryl dioic acid precursor. QY assessments indicated optimal conditions for synthesizing ARI-NCDs which involved combining 15 mg (0.06 mmol) of (*E*)-2-(2,5-dimethoxyphenyl)­methylenebutane-1,4-dioic acid, 0.2 g (1.0 mmol) of CA, and 0.4 g (6.7 mmol) of urea in distilled water (5 mL), followed by hydrothermal treatment at 200 °C for 10 h. After completion of the reactions, the resultant CDs were purified by dialysis against distilled water using a dialysis membrane (MWCO: 1000 Da) for 48 h to eliminate low-molecular-weight impurities. Comprehensive characterization studies focused primarily on novel ARI-NCDs, with comparative evaluations involving U-NCDs. Cellular uptake and cytotoxicity studies were specifically targeted toward ARI-NCDs. Given that U-NCDs derived solely from CA and urea have been reported extensively, the primary aim was to investigate structurally distinct ARI-NCDs for their potential in bioimaging and other biomedical applications.

### 3.2 Characterization of ARI-NCDs and U-NCDs

High-resolution transmission electron microscopy (HR-TEM) was employed to characterize the topography and particle size distribution of the synthesized CDs. As shown in the TEM images (Figure 1a), the ARI-NCDs displayed a uniform spherical shape with a relatively narrow size distribution. The distribution exhibits a right-skewed profile (inset, Figure 1a) indicating a higher concentration of smaller particles in the sample, with a gradual decrease in numbers as the particle size increased. The majority of the particles were clustered around a range of 1.5–3.5 nm, with an average size (μ) = 2.32 ± 1.32 nm. This indicates that most of the particles were fairly close to the average size, suggesting that the distribution of the ARI-NCDs is narrowly distributed. The HR-TEM image (Figure 1b) further revealed regions of pronounced crystallinity in the CDs, distinguished by visible lattice fringes (inset, Figure 1b). Figure 1c,d presents the images at different magnifications.

**Figure 1:**
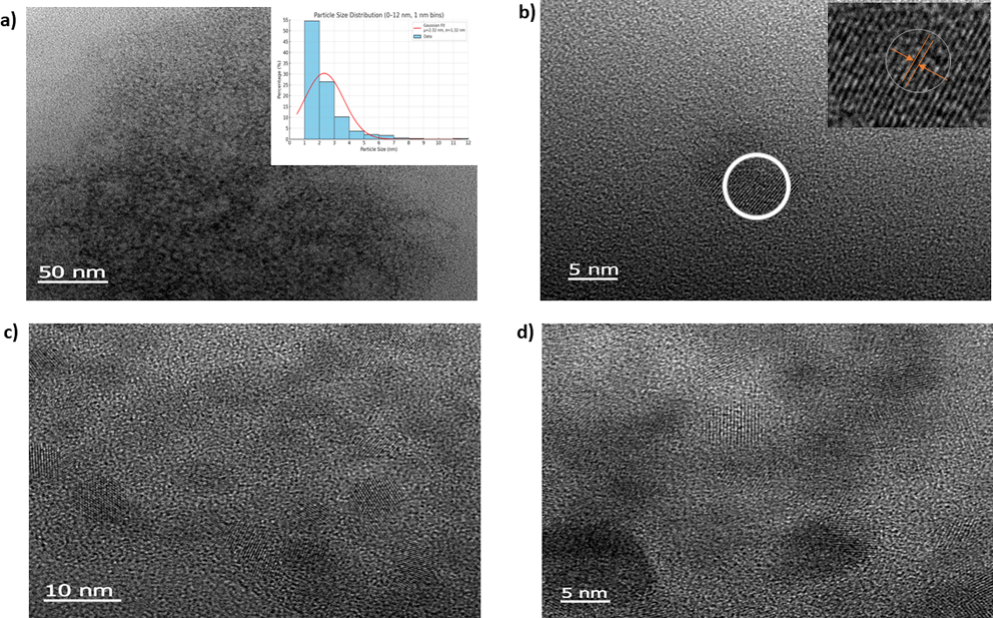
(a) TEM image of ARI- NCDs (inset: size distribution histogram of ARI-NCDs). (b) HR-TEM image (inset: high-resolution lattice fringes). (c, d) TEM images obtained at different magnifications.

FT-IR spectroscopy was employed to investigate the functional groups and chemical structures of both precursor molecules and synthesized CDs. Figure 2a presents the IR spectra of CA, urea, (*E*)-2-(2,5-dimethoxyphenyl)­methylenebutane-1,4-dioic acid, and the synthesized ARI-NCDs, while Figure 2b illustrates the comparative spectra of U-NCDs and ARI-NCDs. The FT-IR spectrum of ARI-NCDs displays a broad absorption band at approximately 3429 cm^–1^, while in U-NCDs, it appears to be slightly shifted at 3417 cm^–1^. This band corresponds to the O–H stretching vibrations of surface-bound hydroxyl and carboxyl groups (−OH), as well as residual moisture. A distinct absorption band centered at 3195 cm^–1^ in ARI-NCDs and at 3182 cm^–1^ in U-NCDs is attributed primarily to N–H stretching vibrations arising from amine (−NH_2_) or amide (−CONH) groups introduced via urea doping during synthesis, with minor contributions from O–H vibrations of carboxyl groups.^35^ These functional groups are commonly observed on the surface of CDs and contribute to their hydrophilic nature.^36^ Notably, ARI-NCDs exhibit additional absorption bands at 2922 and 2852 cm^–1^, attributed to C–H stretching vibrations of alkyl chains or sp^3^-hybridized carbon atoms, possibly resulting from an incomplete carbonization of aryl itaconic acid. These bands are absent in U-NCDs, indicating structural differences related to the presence of an aromatic precursor. A strong band at 1706 cm^–1^ observed in ARI-NCDs is absent in U-NCDs, where absorptions in this region are at 1661 cm^–1^. The absorption in the range of 1720–1650 cm^–1^ is associated with the C=O stretching vibrations of carboxyl (−COOH), amide (−CONH−), or carbonyl groups such as ketones and aldehydes, all commonly found in CDs. Furthermore, the absorption at 1595 cm^–1^ in ARI-NCDs, slightly shifted to 1572 cm^–1^ in U-NCDs, corresponds to N–H bending vibrations from amine or amide groups, also encompassing H–O–H bending from residual moisture. The ARI-NCD spectrum features a band at 1401 cm^–1^ due to O–H bending vibrations, shifted to 1404 cm^–1^ in U-NCDs, indicative of surface hydroxyl functionalities. The unique absorption at 1360 cm^–1^ in ARI-NCDs, assigned to C–N stretching vibrations, signifies amine or amide linkages formed specifically during the reaction involving the 2,5-dimethoxyaryl itaconic acid precursor; this band is notably absent in U-NCDs. The absorption at 1279 cm^–1^ corresponds to C–O stretching vibrations arising from carboxyl (−COO) or ether (−C–O–C) groups.^37^

**Figure 2:**
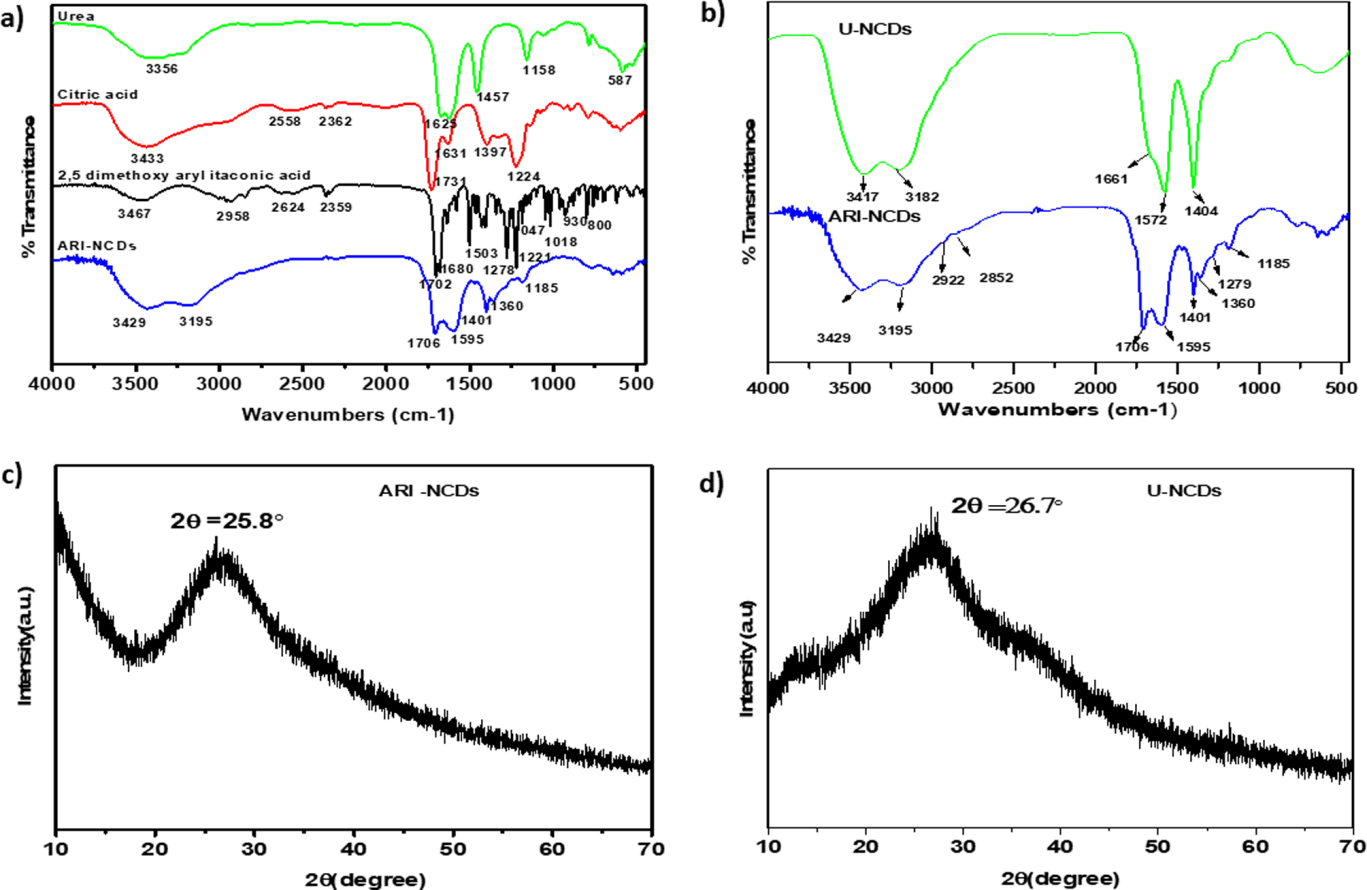
(a) FT-IR spectra of ARI-NCDs and the precursors urea, CA, and (*E*)-2-(2,5-dimethoxyphenyl) methylenebutane-1,4-dioic acid. (b) FT-IR spectra of ARI-NCDs and U-NCDs. (c) XRD pattern of ARI-NCDs. (d) XRD pattern of U-NCDs.

A comparative FT-IR analysis of ARI-NCDs, U-NCDs, and their precursor molecules reveals distinct spectral differences, confirming the successful conversion of precursors to structurally diverse CDs. Both the synthesized CDs possess characteristic hydrophilic functional groups, including hydroxyl, amine, carbonyl, and carboxyl moieties, which are crucial for their aqueous stability and surface reactivity. However, the observed spectral variations clearly demonstrate structural distinctions attributable to the incorporation of the aromatic precursor, significantly influencing the chemical characteristics.

The XRD patterns of ARI-NCDs and U-NCDs are displayed in Figure 2c,d, respectively. Both the diffraction patterns reveal broad, amorphous peaks characteristic of CDs, indicating a predominantly amorphous carbon structure with minor graphitic crystallinity. The ARI-NCDs exhibit a broad diffraction peak centered at approximately 2θ = 25.8° corresponding to the (002) diffraction plane of graphitic carbon.^38^ Similarly, the U-NCDs demonstrate a diffraction peak at 2θ = 26.7°, also ascribed to the (002) plane of the graphitic domains. These broad peaks confirm the partial graphitic crystallinity and the substantial amorphous nature of both sets of synthesized CDs.^39^ The slight shift in the diffraction peak positions between ARI-NCDs and U-NCDs (25.8 vs 26.7°, respectively) may suggest differences in the interlayer spacing of graphitic domains, potentially due to variations in surface functionalization and nitrogen doping resulting from the different precursor compositions. The presence of the aromatic precursor (2,5-dimethoxy aryl itaconic acid) in the ARI-NCD synthesis likely introduces additional structural complexities and surface functionalities, slightly altering the degree of carbonization and crystalline ordering compared to U-NCDs. Overall, the XRD analysis corroborates the successful synthesis of structurally similar yet distinct amorphous CDs, highlighting the subtle structural differences influenced by precursor composition, which may ultimately affect their physicochemical properties and potential applications.

The elemental composition and surface chemistry of ARI-NCDs and U-NCDs were comprehensively analyzed using XPS. Survey spectra for both CDs exhibited clear peaks corresponding to carbon (C 1s), nitrogen (N 1s), and oxygen (O 1s), confirming effective nitrogen doping and surface functionalization (for ARI-NCDs, Figure 3a; for U-NCDs, Figure 3e). The XPS survey spectra revealed characteristic peaks at binding energies of approximately 285 eV (C 1s), 400 eV (N 1s), and 532 eV (O 1s). Quantitative analysis indicated variations in elemental composition between the two CD types, with ARI-NCDs containing 62.1% carbon, 11.0% nitrogen, and 26.9% oxygen and U-NCDs exhibiting 57.3% carbon, 11.6% nitrogen, and 31.1% oxygen. These elemental differences could suggest both slightly different core structures as well as potentially distinct surface chemical environments, which could significantly influence the functional properties of the respective CDs. The high-resolution C 1s spectrum of ARI-NCDs (Figure 3b) can be deconvoluted into several peaks attributed to various functional groups: graphitic sp^2^ carbon (C=C, 284.4 eV), C–C (285.0 eV), C–O–C/C–N–C (285.8 eV), C–OH (286.4 eV), epoxy groups (287.2 eV), carbonyl groups (C=O, 287.9 eV), carboxyl or amide groups (O–C=O/N–C=O, 288.2 eV), and carboxylic groups (O–C–OH, 289.3 eV). Similarly, the C 1s spectrum of U-NCDs (Figure 3f) shows the corresponding functional groups, although the epoxy component observed in ARI-NCDs is notably absent, indicating a slightly different functionalization route during synthesis. The N 1s spectra for ARI-NCDs (Figure 3c) exhibit peaks at 398.5 eV (pyridinic or iminic nitrogen, C–N=C), 399.3 eV (amide or amine groups, NC=O/C–NH_2_), 400.2 eV (amino nitrogen, C–NH−), and 400.5 eV (graphitic or quaternary nitrogen, C3N/C–N+). In contrast, the N 1s spectrum of U-NCDs (Figure 3g) shows fewer nitrogen species, predominantly pyridinic nitrogen (C–N=C, 398.5 eV), amide or amine nitrogen (NC–O/C–NH2, 399.7 eV), and amino nitrogen (C–NH–, 400.8 eV). The presence of quaternary nitrogen exclusively in ARI-NCDs, together with the additional aromatic groups introduced by the aryl precursor, extends the π conjugation and tunes the surface states, thereby modulating their optical and electronic properties, which are attributed to their additional aromatic functionalities from the aryl precursor. The O 1s spectra for both ARI-NCDs and U-NCDs (Figure 3d,h) show characteristic peaks corresponding to carbonyl groups (C=O, 531.0 eV), hydroxyl/carboxyl groups or ethers (C–OH/C–O–C, 532.4–532.5 eV), and carboxylic acid groups (O=C–OH, 533.5 eV). A higher relative intensity of hydroxyl/carboxyl groups in ARI-NCDs compared with U-NCDs suggests a more oxidized surface in ARI-NCDs, potentially leading to enhanced solubility and interaction with biological entities. Collectively, the XPS results reveal notable differences between ARI-NCDs and U-NCDs regarding their surface functionalization and nitrogen doping levels. The richer functionalization in ARI-NCDs, attributed to the aromatic itaconic precursor, likely imparts distinct photophysical and biochemical interactions, which may be beneficial for targeted biomedical applications.

**Figure 3:**
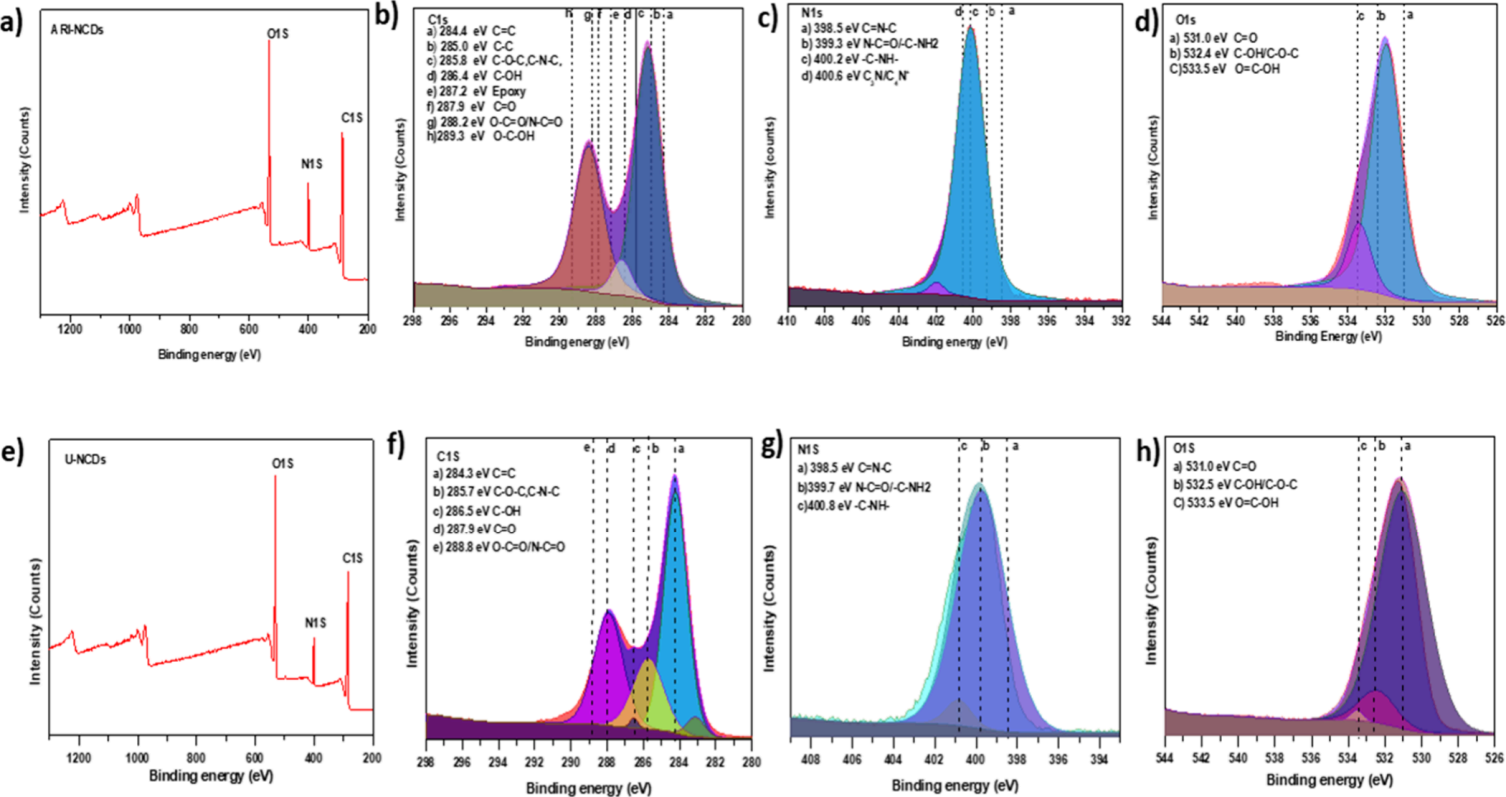
(a) Survey spectrum of ARI-NCDs shows the prominent peaks of C 1s, N 1s, and O 1s. High-resolution (b) C 1s spectrum, (c) N 1s spectrum, and (d) O 1s spectrum of ARI-NCDs. (e) Survey spectrum of U-NCDs. (f) C 1s spectrum, (g) N 1s spectrum, and (h) O 1s spectrum of U-NCDs.

Besides, the obtained ARI-NCDs were analyzed using Raman spectroscopy. Figure 4c presents the Raman spectrum recorded in the spectral range of υ̃ = 500–2200 cm^–1^, along with the corresponding deconvolutions performed using Lorentzian components. Two primary vibrational modes were observed at approximately υ̃ = 1580 and 1360 cm^–1^ corresponding to the G and D bands, respectively.^40^ The broad nature of these bands indicates the presence of a significant number of structural defects within the graphitic or graphene-like framework. The G band primarily arises from the vibrations of sp^2^-hybridized carbon atoms, whereas the D band is a disorder-induced mode activated by symmetry breaking at defects and edges, which is associated with sp^3^-hybridized carbon species. Generally, the intensity ratio of the D to G bands (*I*_D_/*I*_G_), calculated from the integrated intensities of the deconvoluted bands, is known to increase with a decrease in the nanoparticle size due to edge effects. In this study, the *I*_D_/*I*_G_ ratio was determined to be 0.62, suggesting a predominantly amorphous structure of the CDs with some retained graphitic-like structural order. The spectrum implies a moderate disorder in the material, with a mixture of sp^2^ and sp^3^ carbon and possibly oxygen-functionalized groups contributing to the defect structure.

**Figure 4:**
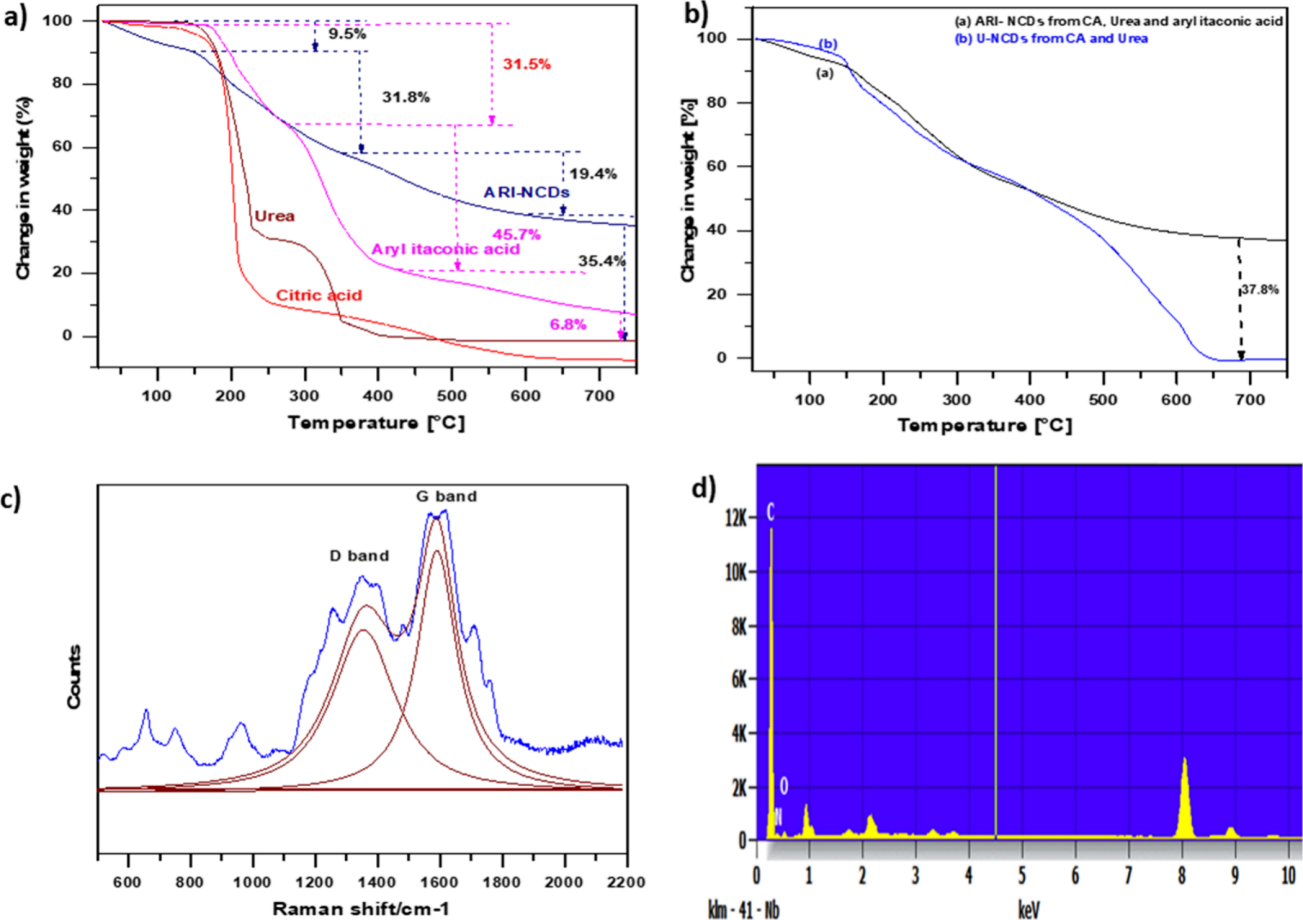
(a) TGA profiles showing weight losses of ARI-NCDs, (*E*)-2-(2,5-dimethoxyphenyl)­methylenebutane-1,4-dioic acid, urea, and CA. (b) Comparative TGAs of ARI-NCDs and U-NCDs. (c) Raman spectrum of ARI-NCDs with deconvoluted Gaussian components; the blue line represents the experimental curve, and the red-purple curves correspond to the Gaussian fits. (d) EDX spectrum of the residue obtained from ARI-NCDs at 750 °C, drop-cast on a copper substrate, and sputter-coated with a gold target. The characteristic Cu Lα, Kα, and K_β_ lines (0.93, 8.04, and 8.91 keV) originate from the copper film, whereas the Au signal (2.12 keV) corresponds to the sputtered gold coating.

TGA of the synthesized CDs, both ARI-NCDs and U-NCDs, was conducted to examine their thermal stability and decomposition behavior in comparison to their precursor molecules.^41^ The TGA profile of ARI-NCDs (Figure 4a) exhibited four distinct decomposition stages: 25–145 °C (loss of moisture and of very volatile components), 145–350 °C (decomposition of oxygen- and nitrogen-containing functional groups), 350–590 °C (breakdown of residual carbon-bonded organic fragments), and 590–750 °C (formation of a stable carbon core). TGA studies of the precursors CA and urea have been reported previously, while (*E*)-2-(2,5-dimethoxyphenyl) methylenebutane-1,4-dioic acid (Figure S3) follows an entirely different decomposition pattern with distinct degradation steps related to moisture loss, decarboxylation, functional group breakdown, and aromatic ring decomposition. The TGA results confirm that the prepared ARI-NCDs possess a reasonably high thermal stability with a 35.4% residual mass content at 750 °C, indicating the formation of a robust core, likely due to the incorporation of the material from (*E*)-2,5 dimethoxyphenylmethylenebutane-1,4-doic acid. The mass losses of the ARI-NCDs in the early stages of TGA correspond to the breakdown of hydrophilic surface groups (−OH, −COOH, and −NH_2_), while the later stages signify structural transformation into a more ordered carbon framework. Although the residual mass content at 750 °C seems excessive, TGAs of CDs including those derived from ternary systems have given similar residual mass content percentages.^42^ In contrast, U-NCDs (Figure 4b), synthesized solely from CA and urea, showed a more gradual decomposition profile with a higher total weight loss and a much less residue at 750 °C. This substantial degradation suggests the formation of less condensed carbon structures and a lower degree of carbonization compared to ARI-NCDs. The comparative TGA results between ARI-NCDs and U-NCDs underscore the influence of aryl itaconic acid as a precursor, contributing to an enhanced carbon content and thermal robustness in ARI-NCDs. This is consistent with the formation of more graphitized or aromatic domains in ARI-NCDs, favorable for applications requiring thermal durability, such as in biomedical formulations and optoelectronic materials. The SEM image (Figure S4a–d) and EDX analysis (Figure 4d) of the residue obtained at 750 °C gives the elemental composition of carbon (48.5%), nitrogen (29.8%), and oxygen (21.7%). These results point to an effective incorporation of nitrogen into the core of the ARI-NCDs.^43^ The high oxygen content of the residual material may be due to the stable ether moieties.

The optical properties of the synthesized ARI-NCDs and U-NCDs were investigated using UV–vis absorption and PL spectroscopies. As shown in Figure 5a, the UV–vis spectrum of the prepared ARI-NCDs exhibits two distinct absorbance peaks at λ = 235 and 330 nm corresponding to the π–π* transition of the aromatic C=C bond present in the graphitic sp^2^ carbon core and the n−π* transition of the C=O bond, respectively.^44^ The sharp absorption peak is typically an indicator of specific electronic transitions and provides insight into the structure, surface states and optical properties of the material.^45^ Many CDs exhibit bright blue fluorescence, a property that underpins their widespread applicability in sensing, imaging, and optoelectronic applications. Figure 5b illustrates the Stokes-shifted PL emission of the prepared ARI-NCDs, with PL measurements revealing an excitation wavelength (λ-_ex_) of 378 nm and a corresponding emission wavelength (λ-_em_) of 445 nm, indicative of strong fluorescence behavior.

**Figure 5:**
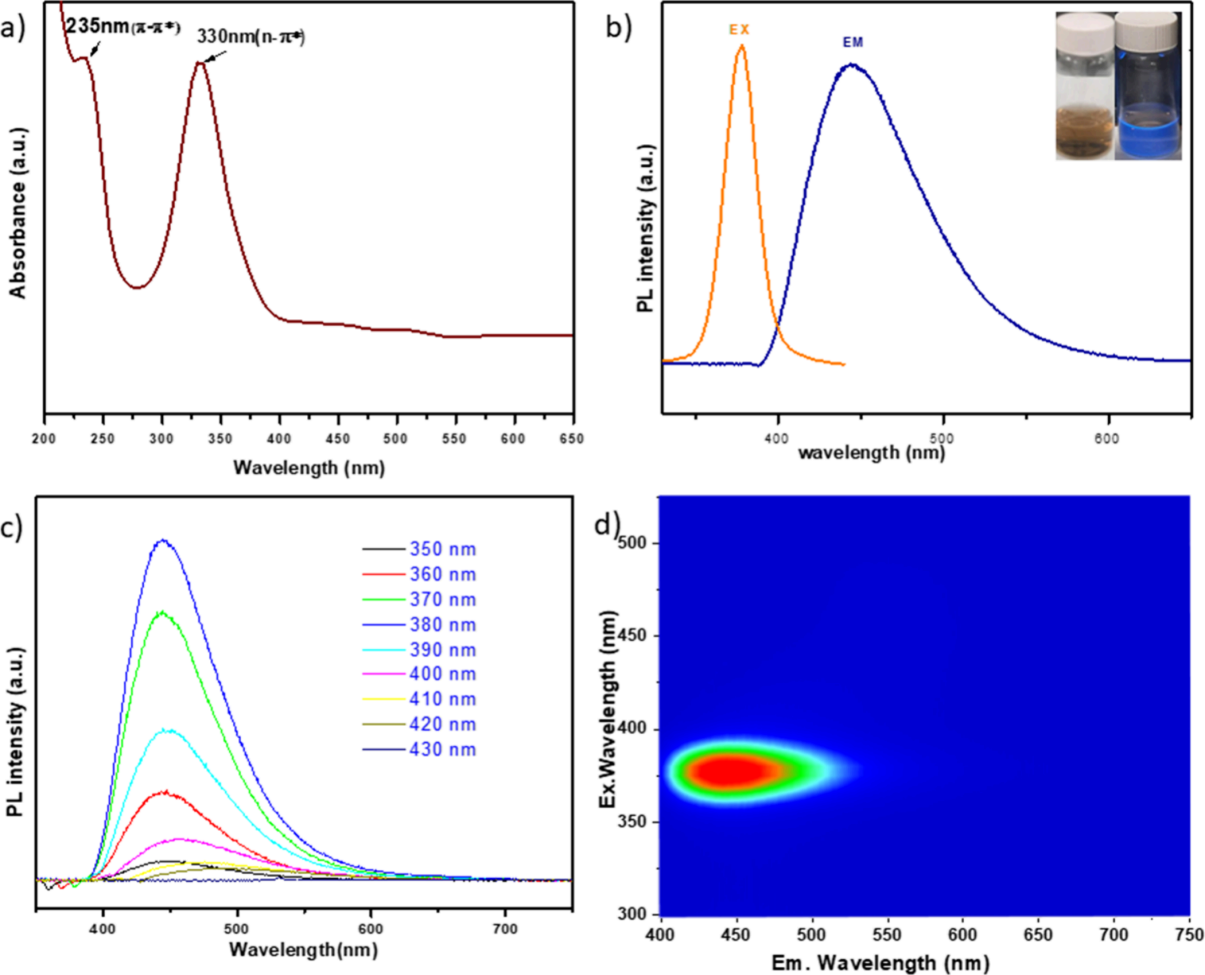
(a) UV–vis absorption spectra of ARI-NCDs. (b) Excitation and emission spectra of ARI-NCDs; the inset shows photographs of ARI-NCDs under daylight (left) and under UV light (λ = 365 nm, right). (c) PL emission of ARI-NCDs recorded at excitation wavelengths ranging from λ = 350 to 430 nm in 10 nm increments. (d) Three-dimensional excitation–emission intensity plot of the synthesized ARI-NCDs.

Upon varying the excitation wavelength (λ-_ex_) from 350 to 430 nm, the PL intensity (Figure 5c) initially increases, reaches a maximum, and then gradually decreases. Notably, the emission peak remains centered at approximately λ-_em_ = 445 nm, regardless of the excitation wavelength. This excitation-independent behavior suggests the presence of uniform and homogeneously distributed sp^2^ cluster domains within the CDs, contributing to consistent emission characteristics.^46^ Adding to that, the CDs appeared light brown under visible light and exhibited bright blue fluorescence under UV irradiation, as depicted in the inset in Figure 5b. The three-dimensional fluorescence map (Figure 5d) further confirms a single, intense excitation band centered at λ = 380 nm and a strong emission peak at λ = 445 nm, reinforcing the observation of stable and excitation-independent PL behavior of the material.

The UV–vis absorption spectra of both ARI-NCDs and U-NCDs (Figure S5a) show nearly identical features, with two distinct absorption bands observed at λ = 235 and 330 nm, while the PL behaviors of ARI-NCDs and U-NCDs exhibit differences. The PL spectra of U-NCDs (Figure S5b) show a strong emission peak centered at λ = 440 nm (vs 445 nm for ARI-NCDs) upon excitation at λ = 364 nm. The difference could be attributed to the simpler precursor system used in U-NCDs, leading to a narrower distribution of emissive states. The incorporation of the aryl-substituted itaconic acid derivative in ARI-NCDs influenced the optical behavior of the CDs. ARI-NCDs exhibit enhanced emission intensity, likely due to increased π- conjugation and the introduction of additional surface functionalities from the 2,5-dimethoxyphenylitaconic acid precursor. This leads to the formation of multiple surface emissive states, offering tunability in fluorescence, a desirable trait for applications such as bioimaging and sensing.

The PL QY of the synthesized ARI-NCDs was determined using eq 1 as described in the Section 2 and was found to be 34.6%. In comparison, U-NCDs synthesized under identical hydrothermal conditions (200 °C for 10 h) exhibited a significantly lower QY of 10.6%. The enhanced QY observed for ARI-NCDs can be attributed to the presence of 2,5- dimethoxyphenyl itaconic acid, which likely promotes improved surface passivation and facilitates the formation of emissive states. Although higher QY values have been reported for CDs derived from CA and urea under optimized or modified synthesis parameters, the present results highlight the beneficial role of incorporating aromatic precursors^37^ in the synthesis to enhance the optical properties of CDs prepared under consistent conditions.

The zeta potential of the synthesized ARI-NCDs and U-NCDs was measured in Milli-Q water and at various buffer conditions (pH 4.0, 7.0, and 10.0) to evaluate their surface charge characteristics and colloidal stability (Figures S6a–d and S7a–d). ARI-NCDs exhibited a zeta potential of −18.9 mV in Milli-Q water, indicating a moderately negative surface charge. At pH 4.0 and pH 7.0, the values were −25.7 and −18.2 mV, respectively, suggesting a predominantly negative surface charge. This is most likely due to the presence of hydroxyl and carboxyl containing functional groups (−COO^–^, −O^–^) on the surface of the CDs.^47^ At alkaline pH (10.0), the ARI-NCDs displayed a complex charge distribution with multiple peaks at −29.9 mV (57.7%), −15.5 mV (21.6%), and −48.8 mV (14.6%), indicating a heterogeneous surface population with diverse charge states.^48^ The observed multicharged peaks at pH 10.0 can be attributed to heterogeneity in functional groups, aggregation effects, and ion adsorption.

In contrast, U-NCDs showed a highly negative zeta potential of −53.9 mV in Milli-Q water. At, pH 4.0, a polydisperse charge distribution was observed with peaks −1.9 and −23.3 mV. At pH 7.0, the U-NCDs also exhibited dual charge species at −40.5 and +5.3 mV, suggesting the presence of coexisting protonated and deprotonated surface functional groups. At pH 10.0, a complex and heterogeneous zeta potential profile was again observed, supporting the presence of diverse functional groups with varying pKa values and multiple charged species with a complex charge distribution.

Although U-NCDs display a stronger negative zeta potential, the moderately negative and tunable surface charge of ARI-NCDs across a wide pH range, particularly their stability and monodisperse distribution near physiological pH, makes them favorable for biological applications. Extremely high surface charges, such as that observed in U-NCDs, can lead to nonspecific protein adsorption, rapid clearance from the bloodstream, or cytotoxicity due to the strong electrostatic interactions with cell membranes.^49^ In contrast, the relatively moderate and tunable negative surface charge of ARI-NCDs facilitates a stable dispersion, reduced aggregation, and minimized nonspecific interactions under physiological conditions. Importantly, at physiological pH (pH 7.32–7.45), corresponding to blood and extracellular fluids, ARI-NCDs maintain colloidal stability and a consistent negative surface charge, which is critical for efficient drug delivery, bioimaging, and biosensing applications.^50^ This pH-responsive surface behavior highlights the potential of ARI-NCDs as versatile and biocompatible nanocarriers in biomedical systems.

### 3.3 Cellular Uptake and Cytotoxicity Studies of ARI-NCDs and U-NCDs

After the successful synthesis and characterization of ARI-NCDs and U-NCDs, we focused our subsequent biological studies on ARI-NCDs as the properties and biocompatibility of U-NCDs have already been extensively reported in the literature.^51–53^ Through cellular uptake and cytotoxicity assessments, we investigated the potential of ARI-NCDs as bioimaging agents. For this purpose, initially, the cellular internalization of ARI-NCDs was studied using HeLa cells as a representative cancer cell.^54^ To monitor the cellular internalization, blue fluorescence of ARI-NCDs was utilized. For this, a solution of ARI-NCDs (0.4 mg/mL) was incubated with HeLa cells for 12 h, and the internalization of the nanoformulation was studied by CLSM analysis. The CLSM images show a strong blue fluorescence signal from the cells, suggesting the effective cellular internalization of ARI-NCDs necessary for bioimaging applications (Figure 6a). Further, to gain deeper insight into the fate of the ARI-NCDs following their cellular entry, colocalization experiments were performed. Lysosomes of the cells were stained using LysoTracker deep red (red fluorescence), and blue fluorescence from ARI-NCDs served to monitor their subcellular localization. Merged CLSM images showed good colocalization of blue fluorescence of ARI-NCDs with the red fluorescence of LysoTracker, suggesting the entrapment of ARI-NCDs at the lysosomes (Figure 6b). The corresponding line analyses also reveal excellent colocalization with a Pearson’s correlation coefficient of 0.45 (Figure 6c).

**Figure 6:**
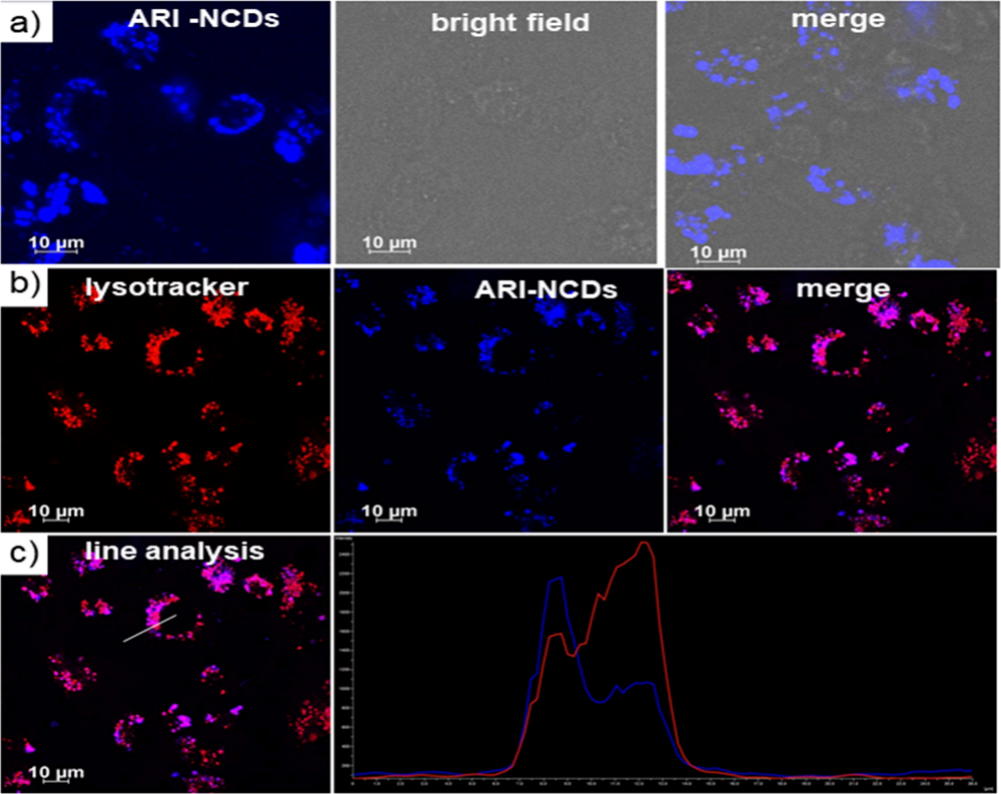
(a) CLSM images of ARI-NCD-treated HeLa cells. (b) Their colocalization at the lysosome and (c) corresponding line analysis representing the colocalization of the ARI-NCDs at the lysosome.

The cellular labeling efficiency of ARI-NCDs compared to U-NCDs was evaluated using HeLa cells incubated with 20 μL (along with 480 μL media) of each nanodot type at a concentration of 0.15 mg/mL for 12 h, followed by CLSM imaging. Under identical imaging settings, HeLa cells treated with ARI-NCDs showed notably higher intracellular fluorescence intensity compared to those treated with U-NCDs, where only weak blue fluorescence was visible (Figure S8a,b). These findings demonstrate that ARI-NCDs possess superior efficiency for cellular labeling over U-NCDs. The enhanced performance of ARI-NCDs can be attributed to the structural and surface chemistry differences imparted by their aryl itaconic acid precursors, which likely promote a greater affinity for cellular membranes and facilitate endocytic internalization. Thus, ARI-NCDs offer clear advantages for bioimaging and potential intracellular delivery applications compared to U-NCDs, primarily due to precursor-derived improvements in their physicochemical interactions with cellular environments.

After demonstrating the efficient cellular internalization of ARI-NCDs followed by lysosomal localization inside the cells,^55^ the in vitro^56^ biocompatibility and cytotoxicity of ARI-NCDs were assessed using HeLa cells treated with varying concentrations of ARI-NCDs (0.1–1 mg/mL) for 24 h, followed by MTT assay^57^ analysis to quantify cell viability. The results demonstrated no significant cytotoxic effects across the entire concentration range, indicating the excellent biocompatibility of ARI-NCDS suitable for bioimaging applications (Figure 7a). Complementary calcein-AM/PI staining corroborated these findings, with HeLa cells treated with both ARI-NCDs and U-NCDs (20 μL from 0.15 mg/mL) exhibiting predominantly green fluorescence, characteristic of viable cells, and the absence of red fluorescence, which would indicate cell death (Figure 7b). Collectively, these data confirm the nontoxic nature of ARI-NCDs, reinforcing their potential for safe biomedical applications, including cellular imaging and drug-delivery modules, without compromising cell viability. These results show that CDs with careful surface functionalization and precursor composition can achieve high biocompatibility while maintaining functional effectiveness in cellular environments.

**Figure 7:**
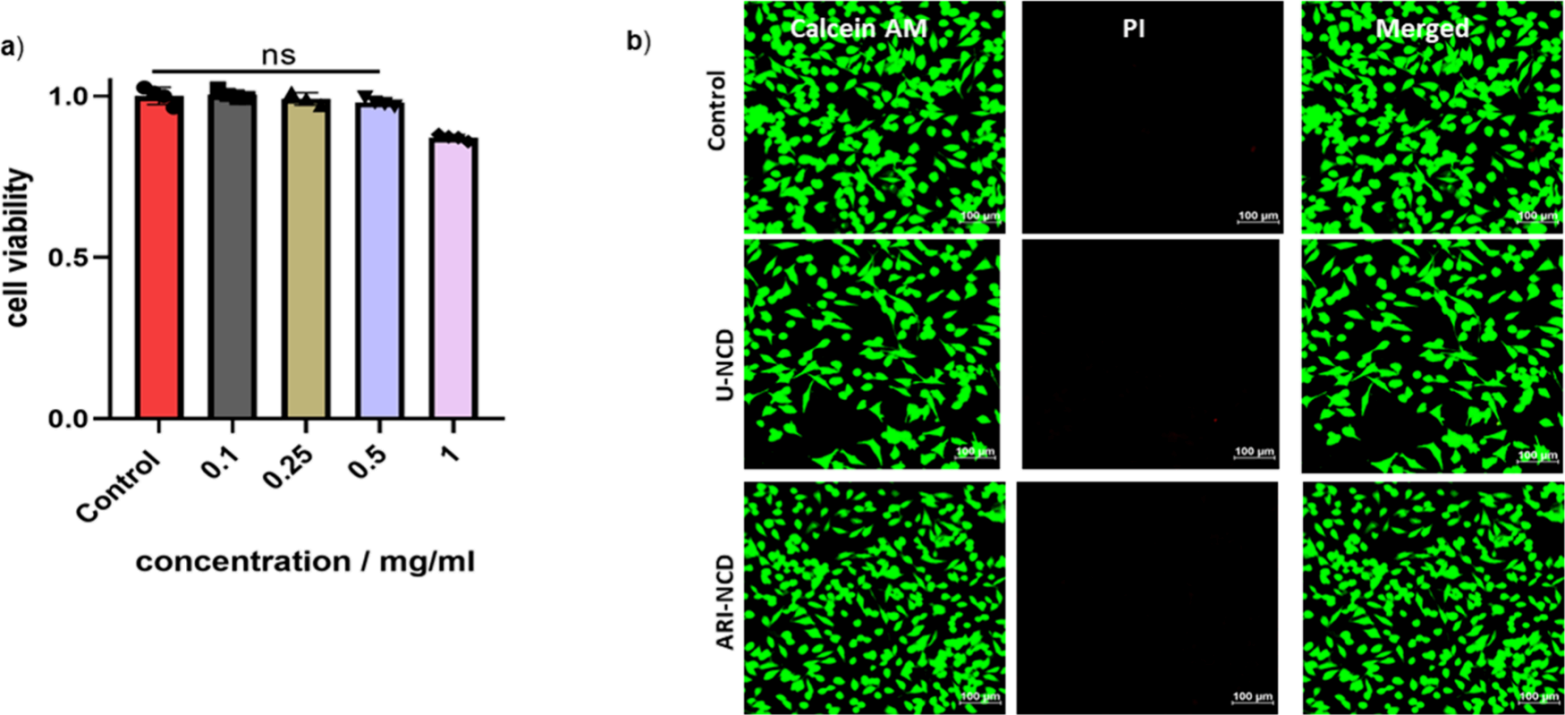
(a) MTT assay with different concentrations of ARI-NCDs (0.1–1 mg/mL) after 24 h. Statistical analyses and data visualization were performed using GraphPad Prism. Comparisons between samples from two groups with normally distributed data with equal variance were made using the unpaired two-tailed Student’s *t* test. For all analyses, *p*-value ≤ 0.05 was accepted as statistically significant. Asterisks in the figures indicate statistical significance ∗ represents *p* < 0.05, ∗∗ represents *p* < 0.01, ∗∗∗ represent *p* < 0.001, and ∗∗∗∗ represent *p* < 0.0001. (b) Calcein-AM/PI live–dead cell assay of U-NCDs- and ARI-NCDs-treated HeLa cells.

### 3.4 Antimicrobial Activity and MIC Assay of ARI-NCDs and U-NCDs

ARI-NCDs demonstrated significantly enhanced antibacterial activity compared with U-NCDs across both Gram-positive and Gram-negative bacterial strains. The inhibition zone diameters for ARI-NCDs ranged from 12 to 16 mm against Gram-positive bacteria, including Bacillus cereus, *Bacillus subtilis*, *Staphylococcus aureus*, and *Staphylococcus lentus* (Table S3 and Figure S9a), consistently exceeding those observed for U-NCDs, which exhibited lower or comparable inhibition, especially against *S. aureus* and *S. lentus*. For Gram-negative bacteria such as **Pseudomonas putida*, *Pseudomonas aeruginosa*, *Klebsiella pneumoniae*,* and *Escherichia coli* , ARI-NCDs produced inhibition zones of 12–15 mm, superior to the 8–10 mm zones observed with U-NCDs (Table S4 and Figure S9b), These results indicate that ARI-NCDs possess stronger antibacterial potential than U-NCDs. Furthermore, MIC assays confirmed that ARI-NCDs achieved effective inhibition at lower concentrations than U-NCDs in several strains (Tables S5 and S6; Figures S10 and S11).

This heightened antimicrobial efficacy of ARI-NCDs is attributed to the incorporation of the (*E*)-2-(2,5-dimethoxyphenyl)­methylenebutane-1,4-dioic acid precursor, which imparts functional groups and structural features that enhance the bacterial membrane interaction and disruption. The presence of aromatic moieties and nitrogen doping may facilitate electron transfer, increase reactive oxygen species generation, and promote binding affinity to bacterial cells, thereby elevating antibacterial performance relative to the U-NCDs. These results underscore the critical role of precursor selection in tuning the biofunctional properties of CDs for antimicrobial applications and highlight ARI-NCDs as promising candidates for further development of antibacterial nanomaterials. The discussion aligns with the observed zones of inhibition and MIC data, clearly illustrating how precursor chemistry determines the antibacterial effectiveness of nitrogen-doped CDs, with ARI-NCDs demonstrating predominant activity over U-NCDs due to their precursor-derived physicochemical advantages.

## 4 Conclusions

In summary, ARI-NCDs were successfully synthesized and characterized using (*E*)-2-(2,5-dimethoxyphenyl)­methylenebutane-1,4-dioic acid, a derivative of itaconic acid, as a precursor molecule in a ternary system, together with urea and CA. Itaconic acid and its derivatives are well-known not only for their anti-inflammatory, antiviral, and anticancer properties but also as versatile platforms for numerous chemical transformations. The synthesized ARI-NCDs were compared with and were found different from U-NCDs prepared under the same conditions but with only CA and urea as precursors. ARI-NCDs were found to exhibit blue and excitation-independent fluorescence. The narrowly distributed particle size distribution of the ARI-NCDs ranges from 1.5 to 3.5 nm. They demonstrate a good thermal stability and an excellent surface charge stability at neutral pH, indicating structural stability at physiological pH values (pH 7.32–7.45). This stability was found to be better than for the U-NCDs and is essential to ensure a reliable performance in drug-delivery systems and other biomedical applications. CLSM analysis confirmed efficient cellular internalization of the prepared ARI-NCDs into HeLa cells. Subsequent colocalization studies further revealed lysosomal entrapment of the ARI-NCDs, evidenced by their excellent overlap with Lysotracker-stained lysosomes and supported quantitatively by a Pearson’s correlation study, leading to a coefficient of 0.45. Extensive biocompatibility assessments, including MTT assays and calcein-AM/PI staining, showed negligible cytotoxicity of the prepared ARI-NCDs toward HeLa cells across a wide concentration range, highlighting the exceptional biocompatibility of the ARI-NCDs. Apart from their excellent physicochemical and biological properties, ARI-NCDs demonstrated significantly enhanced antibacterial activity compared to U-NCDs across both Gram-positive and Gram-negative bacterial strains. Collectively, these findings underscore the significant potential of the ARI-NCDs as safe and effective bioimaging agents for biomedical applications. However, further in vivo investigations are required to fully explore their utility as therapeutic nanotools and to elucidate the influence of the surface chemistry on their performance in drug-delivery systems.

## Supplementary Material



## Data Availability

All data supporting the findings of this study are available within the article and its Supporting Information

## Notes

Synthetic route to (*E*)- 2-(2,5- dimethoxyphenyl)­methylenebutane-1,4-dioic acid, its characterization by ^1^H NMR and ^13^C NMR spectra, and its antibacterial activity against Gram-positive strains *Staphylococcus aureus* and *Staphylococcus lentus* as well as Gram-negative strains *Pseudomonas aeruginosa* and **Pseudomonas putida*;* TGA decomposition profile of the acid, SEM images and elemental mappings of carbon, nitrogen, and oxygen from the residual ARI-NCD sample after thermolysis at 750 °C, UV–vis absorption and excitation/emission spectra of U-NCDs, and zeta potential measurements of ARI-NCDs and U-NCDs in Milli-Q water and at various pH values; CLSM images of the cellular labeling efficiency of ARI-NCDs and U-NCDs; table of sample assay concentration, working concentration, and MIC concentrations of ARI-NCDs and U-NCDs; table of antibacterial activity of ARI-NCDs and U-NCDs against four Gram-positive and Gram-negative pathogens; heat map of Gram-positive and Gram-negative bacterial pathogen activities; MIC assay of ARI-NCDs and U-NCDs against four Gram-positive and Gram-negative bacterial pathogens; and heat map of MIC in millimeters for four Gram-positive and Gram-negative bacterial pathogens across varying concentrations (PDF)
