# Supplementary material for: Design, Synthesis, and Characterization of N‑Doped Carbon Dots from a Ternary System of Citric Acid, Urea, and (E)‑2-(2,5-Dimethoxyphenyl)methylenebutane-1,4-dioic Acid
Source: ACS Omega. 2026 Mar 14;11(11):17235–48. doi: 10.1021/acsomega.5c08957 (PMC13019246; doi:10.1021/acsomega.5c08957)
Supplement: Supplementary file 1 [file ao5c08957_si_001.pdf]

Supplementary Data

Design,Synthesis, and Characterization of N- Doped Carbon Dots from a Ternary System of Citric acid, Urea, and (*E*)-2-(2,5-Dimethoxyphenyl)methylenebutane-1,4-dioic Acid.

Vijo Poulose[a]* ,Keerthivasan M. Latha[b] Gowtham Raj[b], Sabu Thomas[c], Józef Haponiuk T[d], Reji Varghese[b], Thies Thiemann[a], Sreeraj Gopi[e]*

[a] *Department of Chemistry, College of Science, United Arab Emirates University, Al Ain PO Box 15551 United Arab Emirates*

[b] *School of Chemistry, Indian Institute of Science Education and Research (IISER) Thiruvananthapuram, Trivandrum-695551, Kerala, India.*

[c] *School of Nanoscience and Nanotechnology, Mahatma Gandhi University, Kottayam-686560, Kerala, India*

[d] *Department of Polymer Technology, Faculty of Chemistry Gdańsk University of Technology, Gdańsk, Pomeranian Voivodeship 80-233, Poland*

[e] *R&D Centre, Molecules Biolabs Private Limited, Koratty, Thrissur-680309, Kerala, India*

*Correspondence: vijo.p@uaeu.ac.ae, drgopisreeraj@gmail.com

Index

Scheme S1. Synthetic route to (*E*)-2-(2,5-dimethoxyphenyl)methylenebutane-1,4-dioic acid (**5**) p.3

Figure S1. a) ^{1}H NMR spectrum of **5** (395.7 MHz, DMSO-d^6) b) ^{13}C NMR spectrum of **5** (100.5 MHz, DMSO-d^6) p.3

Figure S2. Activity of **5** against Gram positive strains a) *S. aureus* and b) *S. lentus* and Gram-negative strains c) *P. aeruginosa* and d) *P. putida* p.4

Figure S3. TGA decomposition profile of (*E*)-2-(2,5-dimethoxyphenyl)methylenebutane-1,4-dioic acid (**5**) p.4

Figure S4. a)The SEM image and elemental mapping of b) carbon,c) nitrogen and d) oxygen of the residual sample from ARI-NCDs, after thermolysis at 750 °C p.5

Figure S5. a.)UV-Vis absorption spectra of U-NCDs; b.) Excitation and emission spectra of U-NCDs p.6

Figure S6. Zeta potenial measurements of ARI-NCDs in a) milliQ water (-18.9mV) and at different pH values, namely at b) pH 4.0 (-25.7mV), c) 7.0 (-18,2mV) and d) 10.0 (differently charged species) p.6

Figure S7. Zeta potential measurements of U-NCDs in (a) Milli-Q water (−53.9 mV), and at different pH conditions: (b) pH 4.0 showing polydisperse charge species (−1.39 mV, −23.3

mV), (c) pH 7.0 shows multicharged species (−40.5 mV, +5.3 mV), and (d) pH 10.0 indicating differently charged species p.7

Figure S8. The CLSM images of cellular uptake efficiency of a) ARI-NCDs and b) U-NCDs p. 7

Table S1 Sample assay concentration and working concentration p.8

Table S2 Minimum inhibitory Concentrations of ARI-NCDs and U-NCDs p.8

Table S3. Antibacterial activity of ARI-NCDs and U-NCDs against four-gram positive pathogens p.8

Table S4. Antibacterial activity of ARI-NCDs and U-NCDs against four-gram negative pathogens p.8

Figure S9 a**)** Heat map of gram positive bacterial pathogens activity b**)** Heat map of gram negative bacterial pathogens activity p.9

Table S5. Minimum inhibitory concentration (MIC) assay of ARI-NCDs and U-NCDs against four gram-positive bacterial pathogens p.9

Figure S10 Heat map of minimum inhibitory concentration in millimeters for four gram-positive bacterial pathogens across varying concentrations p.10

Table S6. Minimum inhibitory concentration (MIC) assay of ARI-NCDs and U-NCDs against four gram-negative bacterial pathogens p.10

Figure S11 Heat map of minimum inhibitory concentration in millimeters for four gram-negative bacterial pathogens across varying concentrations p.11

.

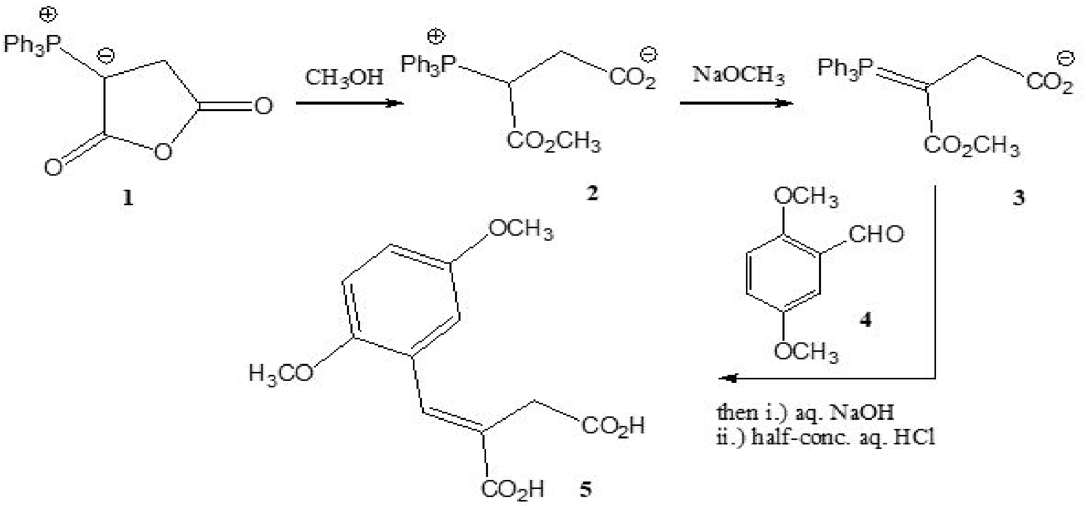

Scheme S1. Synthetic route to (*E*)-2-(2,5-dimethoxyphenyl)methylenebutane-1,4-dioic acid (**5**)

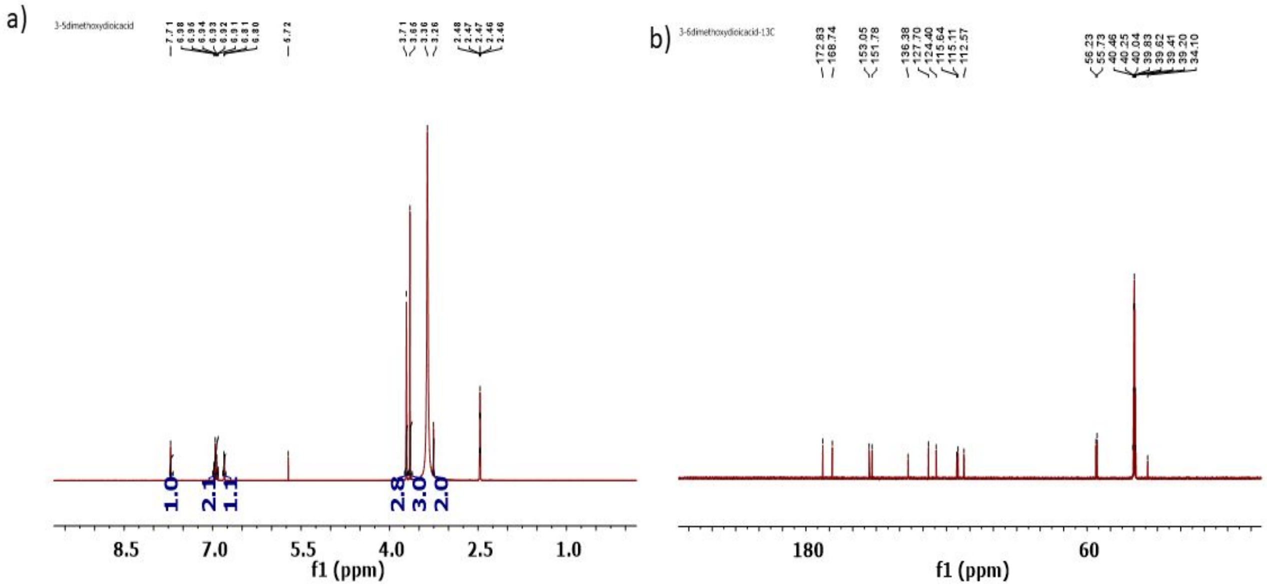

Figure S1. a) ^{1}H NMR spectrum of **5** (395.7 MHz, DMSO-d^{6}) b) ^{13}C NMR spectrum of **5** (100.5 MHz, DMSO-d^{6})

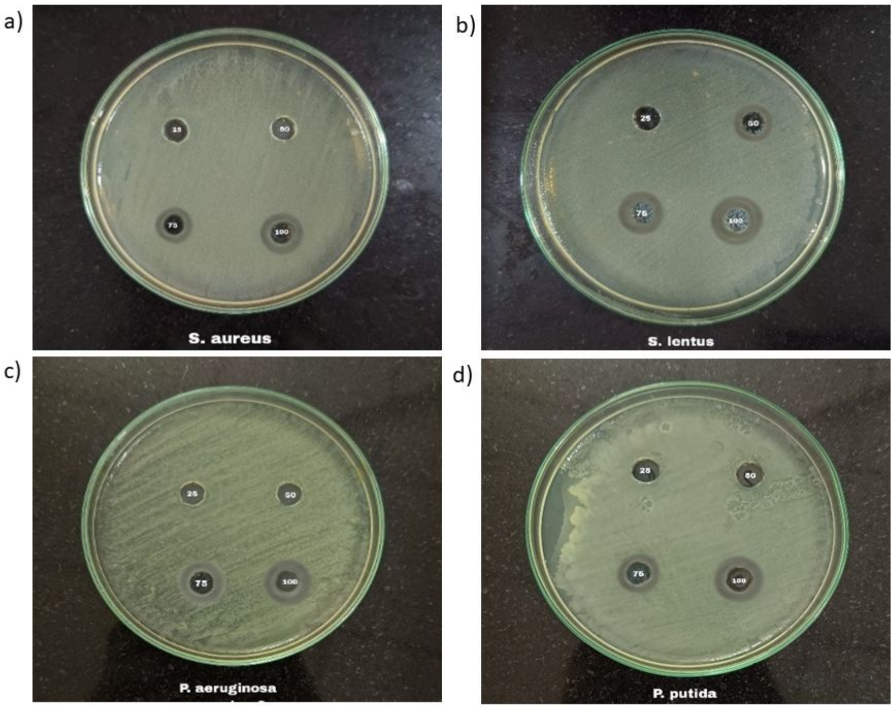

Figure S2. Activity of **5** against Gram positive strains a) *S. aureus* and b) *S. lentus* and Gram-negative strains c) *P. aeruginosa* and d) *P. putida*

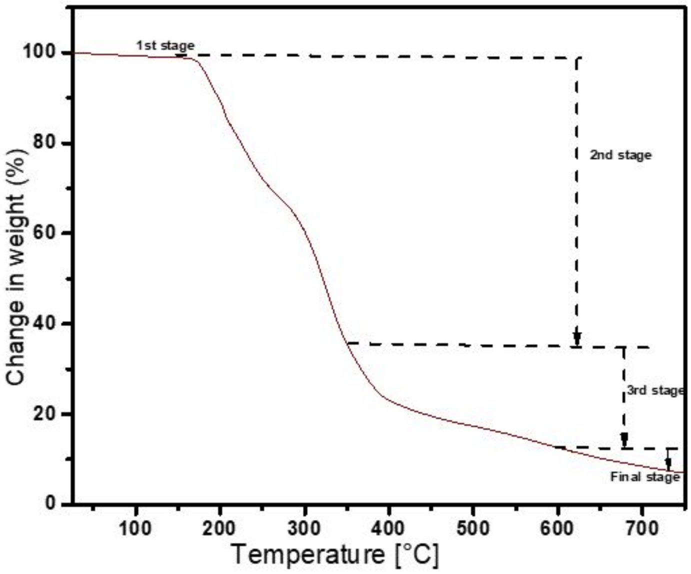

Figure S3. TGA decomposition profile of (E)-2-(2,5-dimethoxyphenyl)methylenebutane-1,4-dioic acid (**5**)

The thermal decomposition of **5** (Figure S3) can be observed to proceed through four distinct stages. The first stage, occurring between 25-145°C, involves the evaporation of moisture and volatile components. The second stage, from 145–350°C, involves chemical decomposition, primarily characterized by decarboxylation. The third stage, between 350–590°C, is marked by the breakdown of further functional groups followed by decomposition of the aromatic ring structure. Finally, the fourth stage, occurring from 590–750°C leads to the formation of residual carbonaceous material.

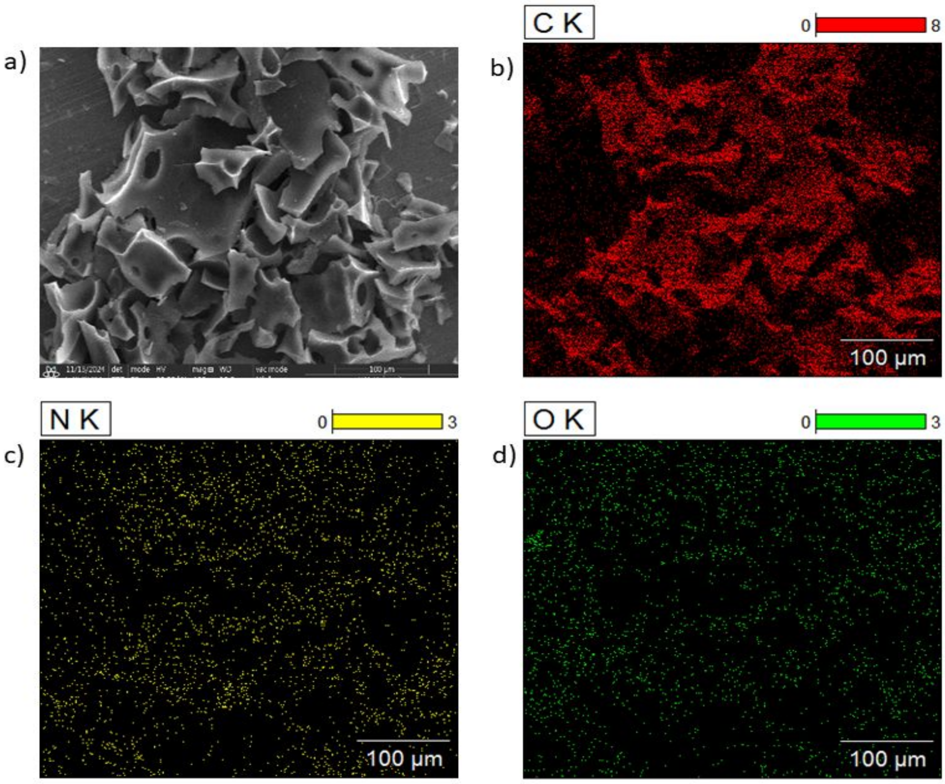

Figure S4. a) The SEM image and elemental mapping of b) carbon,c) nitrogen and d) oxygen of the residual sample, after thermolysis at 750 °C of ARI-NCDs

The Field Emission Scanning Electron Microscopy (FE-SEM) image and elemental mapping of the CD residue obtained at 750°C from ARI-NCDs (Figure S4) confirm the presence of carbon, nitrogen, and oxygen, providing insights into the elemental composition of the carbon core of the ARI-NCDs. However, these results do not directly correlate with the XPS findings**,** as FE-SEM-EDX is a purely destructive analytical technique that only determines elemental distribution without providing information on chemical bonding. Despite this limitation, the data gives further information on the structural composition of the ARI-NCDs and further proves the incorporation of nitrogen and oxygen into the carbon framework.

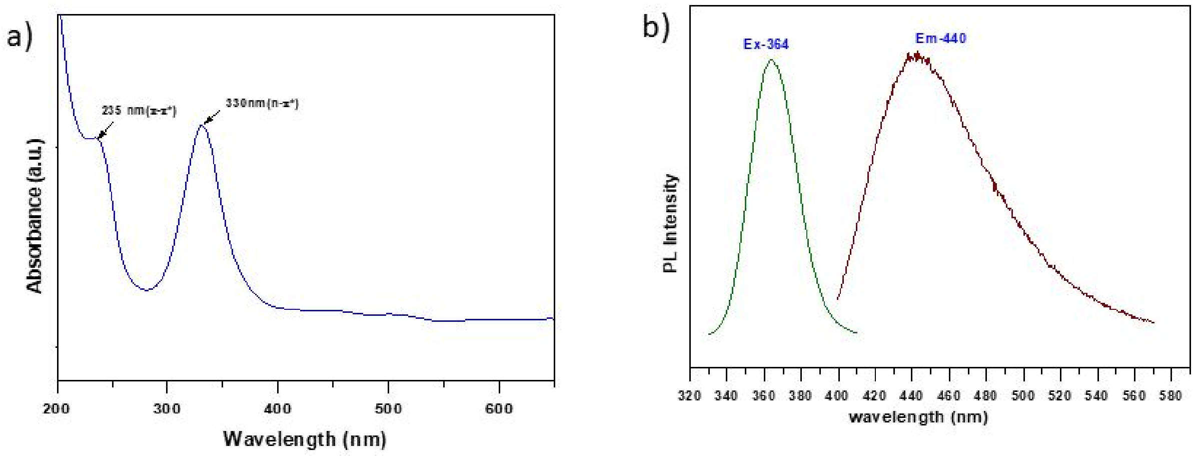

Figure S5. a.)UV-Vis absorption spectra of U-NCDs; b.) Excitation and emission spectra of U-NCDs.

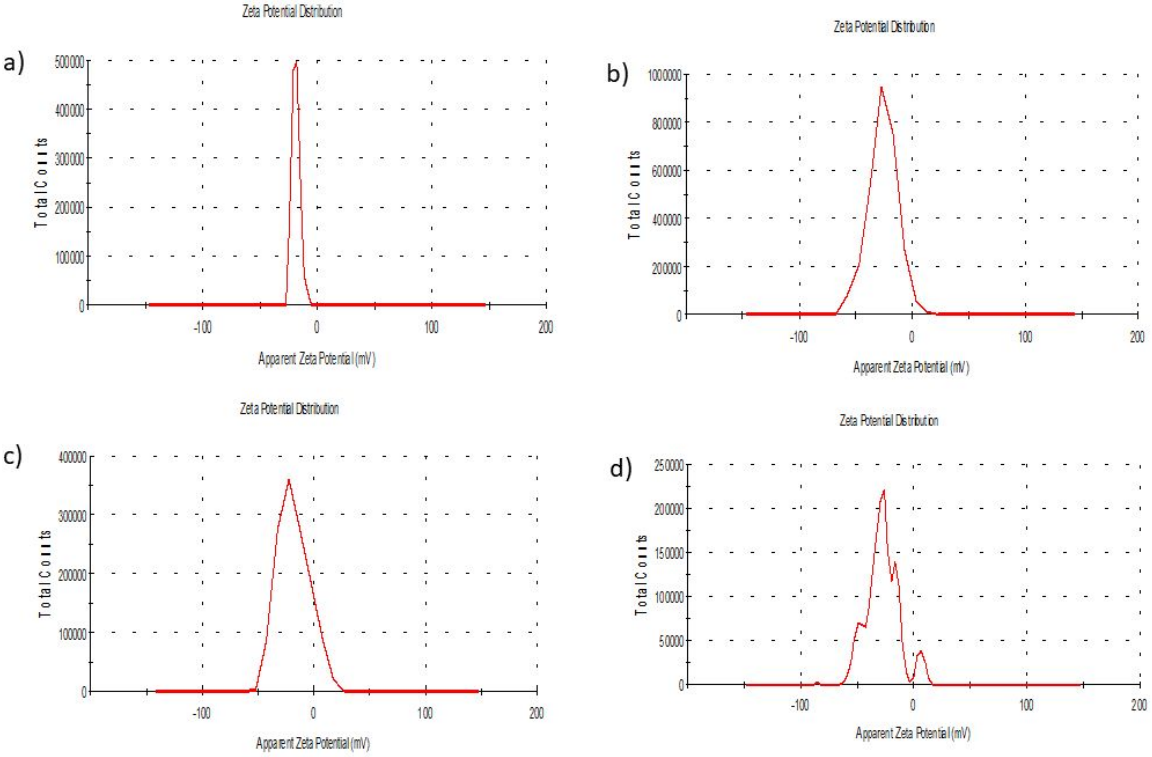

Figure S6. Zeta potenial measurements of ARI-NCDs in a) milliQ water (-18.9mV) and at different buffer conditions, namely at b) pH 4.0 (-25.7mV), c) 7.0 (-18,2mV) and d) 10.0 (differently charged species)

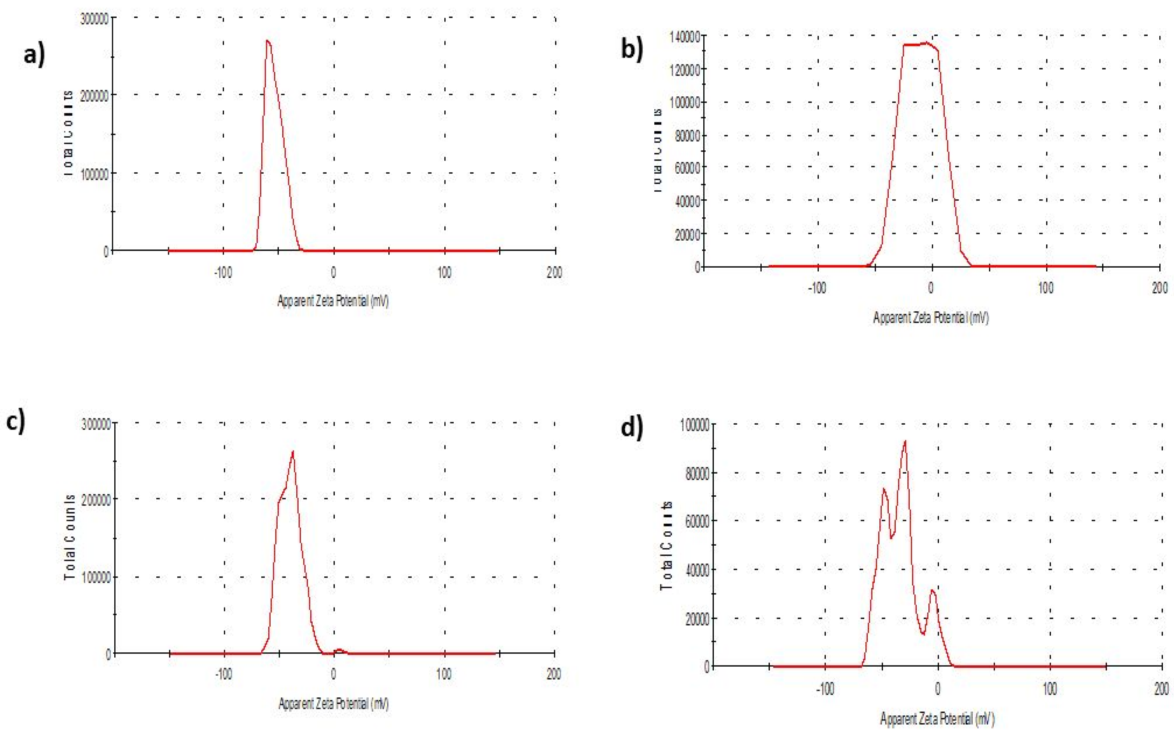

Figure S7. Zeta potential measurements of U-NCDs in (a) Milli-Q water (−53.9 mV), and at different buffer conditions: b) pH 4.0 showing polydisperse charge species (−1.39 mV, −23.3 mV), (c) pH 7.0 shows multicharged species (−40.5 mV, +5.3 mV), and (d) pH 10.0 indicating differently charged species.

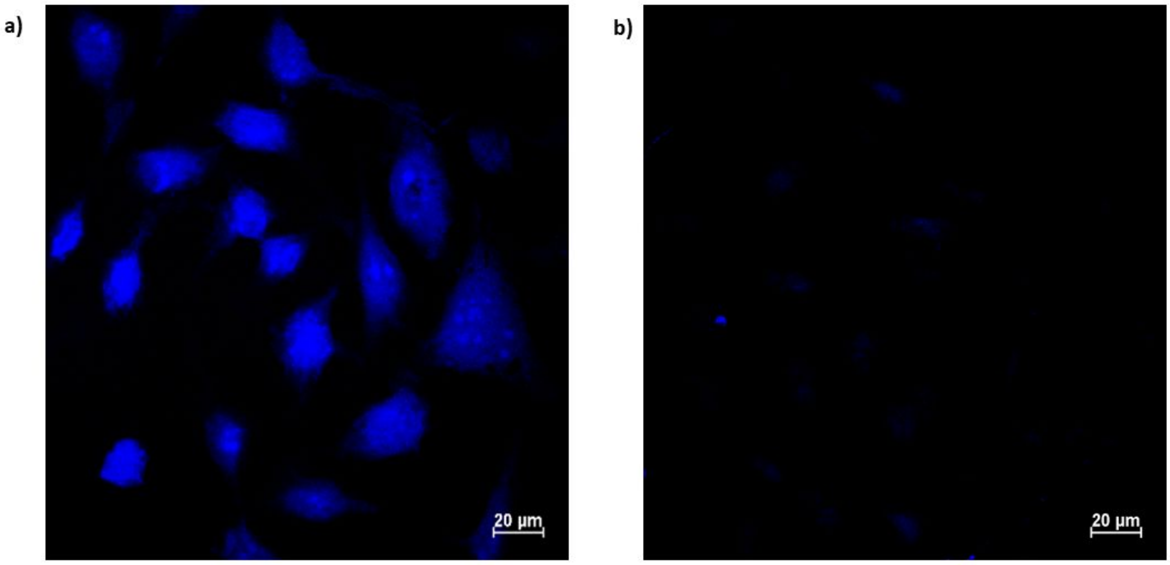

Figure S8 a) The CLSM images of cellular labelling efficiency of ARI-NCDs and b) U-NCDs

The active bacterial cultures prepared by as single bacterial colony of pure culture was transferred into a 150mL conical flask containing 50mL nutrient broth media and incubated for 8-12h at 37°C.The samples were dissolved in 1mL water and made into aliquots of different concentrations for MIC assay.

S. No.	Sample and concentration	Working concentration
1	**ARI-NCDS**<br>1.2mg/mL	300 µg/mL
2	**U-NCDS**<br>1.5mg/mL	375 µg/mL

Table S1 Sample assay concentration and working concentration

Well dilutions	25µL	50µL	75µL	100µL
**ARI-NCDS**	7.50 µg/well	15 µg/well	22.5 µg/well	30 µg/well
**U-NCDS**	9.37 µg/well	18.75 µg/well	28.12 µg/well	37.5 µg/well

Table S2 Minimum inhibitory Concentrations of ARI-NCDs and U-NCDs

S.no	Sample Name	Gram Positive Bacterial pathogens			
		B. cereus	***B. subtilis***	***S. aureus***	***S. lentus***
**1**	**ARI-NCDS**	14mm	12mm	16mm	16mm
**2**	**U-NCDS**	14mm	14mm	10mm	12mm
**3**	**Standard**	>25mm	>25mm	16mm	15mm

Table S3. Antibacterial activity of ARI-NCDs and U-NCDs against four-gram positive pathogens.

S.no	Sample Name	Gram Negative Bacterial pathogens			
		P. putida	***P. aeruginosa***	***K. pneumoniae***	***E. coli***
**1**	**ARI-NCDS**	14mm	15mm	12mm	12mm
**2**	**U-NCDS**	08mm	10mm	10mm	10mm
**3**	**Standard**	16mm	15mm	18mm	16mm

Table S4. Antibacterial activity of ARI-NCDs and U-NCDs against four-gram negative pathogens

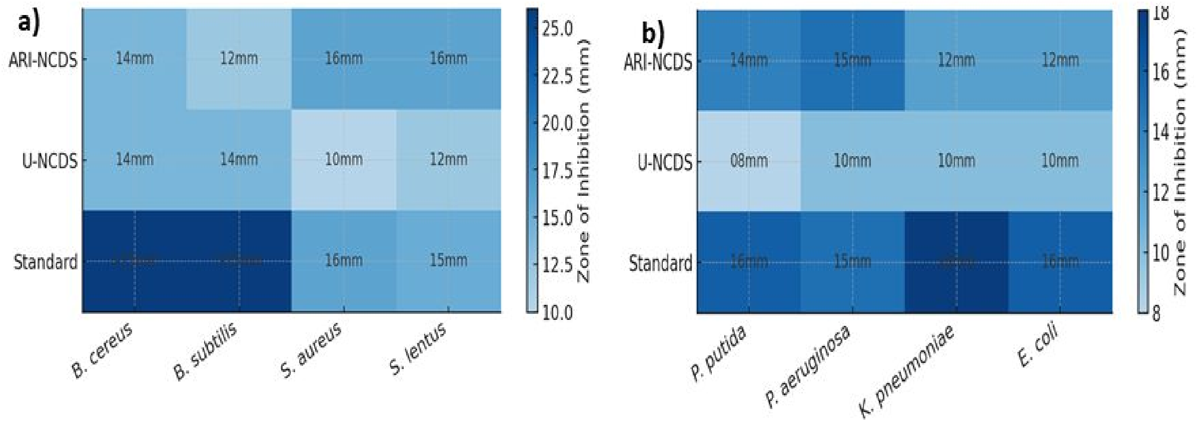

Figure S9 a) Heat map of gram positive bacterial pathogens activity **b)** Heat map of gram negative bacterial pathogens activity

S.no	Sample ID	MINIMUM INHIBITORY CONCENTRATION – MIC (mm)				MIC of sample (µL)
B. subtilis						
		**25µL**	**50µL**	**75µL**	**100µL**	
**1**	**ARI-NCDS**	10mm	10mm	12mm	14mm	**25µL**
**2**	**U-NCDS**	12mm	12mm	12mm	14mm	**25µL**
B. cereus						
**1**	**ARI-NCDS**	16mm	16mm	16mm	16mm	**25µL**
**2**	**U-NCDS**	08mm	10mm	12mm	14mm	**25µL**
S. aureus						
**1**	**ARI-NCDS**	12mm	12mm	12mm	16mm	**25µL**
**2**	**U-NCDS**	08mm	08mm	10mm	12mm	**25µL**
S. lentus						
**1**	**ARI-NCDS**	08mm	10mm	12mm	15mm	**25µL**
**2**	**U-NCDS**	-	08mm	10mm	14mm	**75µL**

Table S5. Minimum inhibitory concentration (MIC) assay of ARI-NCDs and U-NCDs against four gram-positive bacterial pathogens.

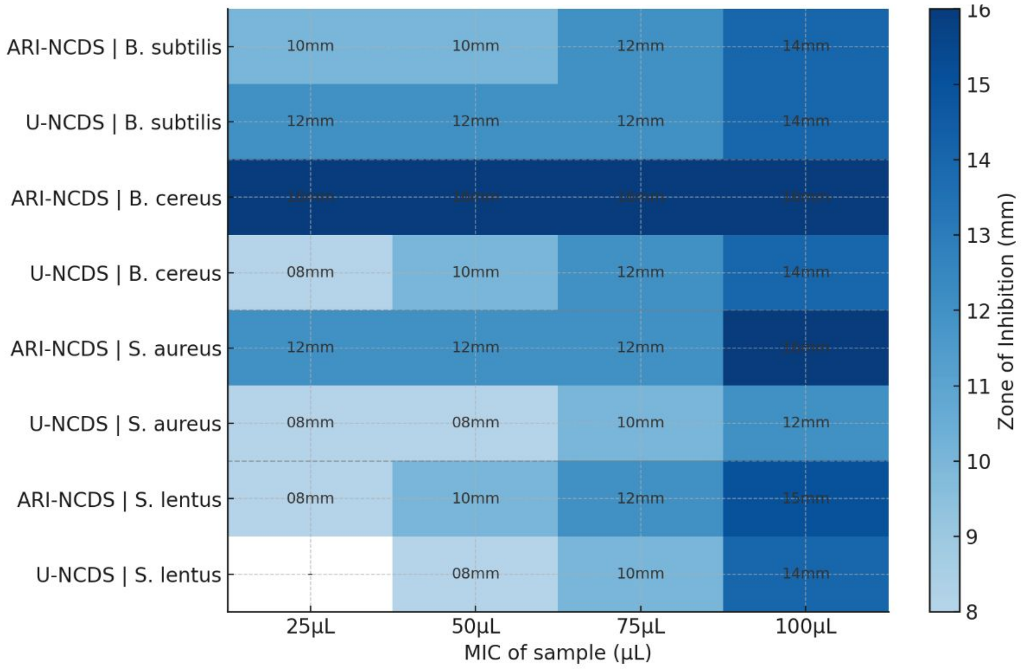

Figure S10 Heat map of minimum inhibitory concentration in millimeters for four gram-positive bacterial pathogens across varying concentrations.

S.no	Sample ID	MINIMUM INHIBITORY CONCENTRATION – MIC (mm)				MIC of sample (µL)
		P. putida				
		**25µL**	**50µL**	**75µL**	**100µL**	
**1**	**ARI-NCDS**	08mm	08mm	10mm	16mm	**25µL**
**2**	**U-NCDS**	-	-	12mm	14mm	**75µL**
		P. aeruginosa				
**1**	**ARI-NCDS**	07mm	07mm	12mm	16mm	**25µL**
**2**	**U-NCDS**	-	-	12mm	14mm	**75µL**
		K. pneumoniae				
**1**	**ARI-NCDS**	-	07mm	10mm	12mm	**50µL**
**2**	**U-NCDS**	-	-	10mm	12mm	**75µL**
		E. coli				
**1**	**ARI-NCDS**	-	-	10mm	12mm	**75µL**
**2**	**U-NCDS**	-	-	10mm	12mm	**25µL**

Table S6. Minimum inhibitory concentration (MIC) assay of ARI-NCDs and U-NCDs against four gram-negative bacterial pathogens.

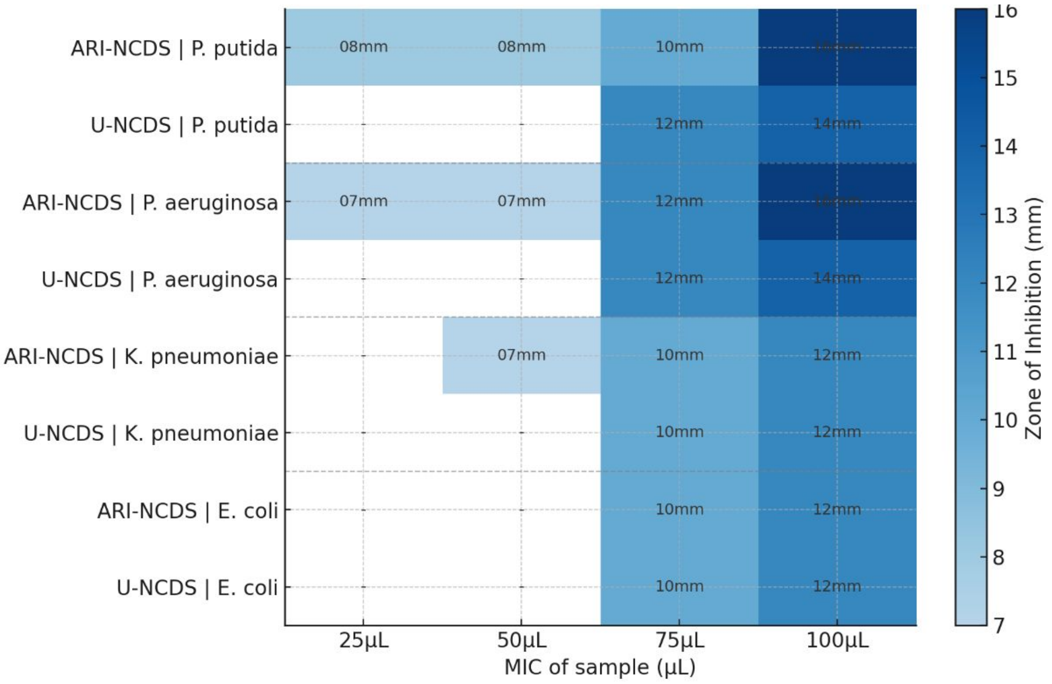

Figure S11 Heat map of minimum inhibitory concentration in millimeters for four gram-negative bacterial pathogens across varying concentrations.
